# Preharvest antibiotic use influences antibiotic resistance in *Salmonella* species from commercial poultry and swine farms in Lagos, Southwestern Nigeria

**DOI:** 10.3389/fmicb.2026.1825884

**Published:** 2026-04-28

**Authors:** Timothy Obiebe Jason Odey, Kayode Olayinka Afolabi, Omotayo Babatunde Komolafe, Williams Omotola Tanimowo, Silas Dogara Gyar, Rine Christopher Reuben

**Affiliations:** 1 Department of Biological Sciences, Faculty of Science, Anchor University, Lagos, Nigeria; 2 Department of Microbiology, Faculty of Science, University of Ibadan, Ibadan, Nigeria; 3 Department of Microbiology, Nassarawa State University, Keffi, Nigeria; 4 Department of Biology, Stephen F. Austin State University, Nacogdoches, TX, United States

**Keywords:** antimicrobial resistance (AMR), antimicrobial stewardship, food animals, multidrug resistance, One Health, preharvest drivers, *Salmonella*

## Abstract

**Introduction:**

The widespread use of antibiotics in commercial food animal production for growth promotion and prophylaxis is a primary driver of antimicrobial resistance (AMR) globally. This study provides a systematic characterization of the drivers of pre-harvest AMR in *Salmonella* spp. across farms in Lagos, southwestern Nigeria.

**Methods:**

We evaluated 3,600 fecal samples from animals on commercial poultry and swine farms for *Salmonella* spp. and for antimicrobial and virulence determinants, using established microbiological, biochemical, and molecular protocols. We further assessed farm management practices and antimicrobial use through structured questionnaires and correlated these data with antimicrobial resistance phenotypes and determinants in *Salmonella* strains recovered from fecal samples across the farms.

**Results:**

Overall, we observed a 1.5% (54/3,600) prevalence of *Salmonella* spp. across the sampled animals, with 0.67, 1.67, and 2.2% in layers, broilers, and pigs, respectively. While most *Salmonella* strains showed multidrug resistance, high rates of erythromycin (80–100%) and tetracycline (75–92.3%) resistance were observed across animal species, with multiple antibiotic resistance indices (MARI) ranging from 0.2 to 0.9. Molecular analysis revealed widespread distribution of major resistance determinants - *tetA* (70–100%), *ant* (3”)-*Ia* (80.8–100%), and *sul1* (65.4–80%), and high prevalence of *Salmonella*-associated virulence factors, including *iroB* (62.5–75%), *pipD* (37.5–92.3%), and *orfL* (65–88.5%). Furthermore, we found extensive preharvest antibiotic use [as growth promoters (53.8–85.7%) and prophylaxis (71.4–81.3%)] as well as weak positive correlations (*r* = 0.058–0.487, *p* = 0.327–0.913) between antibiotic use and AMR phenotypes across animal species.

**Discussion:**

Our study reveals widespread AMR and virulence determinants in *Salmonella* spp. from commercial farms, suggesting a potential public health risk and the need for enhanced investigation, antimicrobial stewardship, and a concerted One Health mitigation strategy.

## Graphical Abstract

**Figure:**
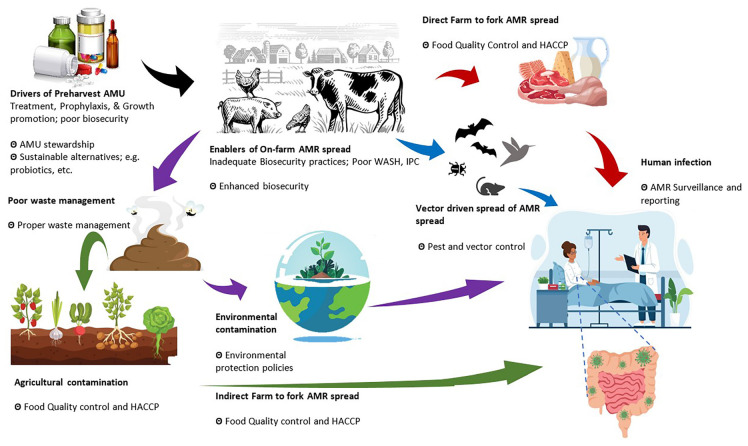
Interconnection of preharvest antimicrobial use (AMU), On-farm biosecurity practices, and waste disposal in the dissemination of Antimicrobial resistance (AMR).

## Introduction

Antimicrobial resistance (AMR) remains one of the most significant challenges to public health, food safety, and sustainable livestock production worldwide (Musuka et al., 2025). The intensification of livestock production to meet rising demand for animal protein in developing economies has led to a heavy reliance on antimicrobials for treatment, prophylaxis, and, in some cases, growth promotion (Elbehiry et al., 2025). The extensive use of antibiotics in food-animal systems has accelerated the selection and spread of resistant bacterial strains within agricultural environments (Elbehiry and Marzouk, 2025). Among the pathogens of greatest concern is *Salmonella*, a leading cause of foodborne illness globally and a zoonotic organism capable of moving between animal reservoirs and human populations (Zizza et al., 2024). The emergence of antibiotic-resistant *Salmonella* in livestock production systems poses a direct threat to treatment efficacy, increases the risk of outbreaks, and contributes to the broader environmental pool of resistance genes (Zheng et al., 2024).

Aggravating this challenge is the increasing prevalence of multidrug-resistant (MDR) *Salmonella* (Lv et al., 2024). Sub-Saharan Africa reports disproportionately high AMR rates, with recent meta-analyses documenting a 68.5% prevalence of multidrug resistance in non-typhoidal *Salmonella* (NTS) human infections (Amir et al., 2024). This elevated resistance stems from widespread preharvest antimicrobial use, including sublethal dosing in feeds for growth promotion and prophylactic applications without veterinary oversight (Odey et al., 2023). These practices are particularly prevalent in West African livestock systems, where regulatory gaps and limited veterinary oversight enable indiscriminate antimicrobial use (Aworh et al., 2024). This burden is perhaps most acutely felt in Nigeria, the region’s most populous nation and a significant livestock-producing economy, where pervasive unsupervised and subtherapeutic antimicrobial use across poultry and ruminant production systems continues to exert selective pressure on bacterial populations, with far-reaching implications for both animal and human health (Daodu et al., 2025; Caneschi et al., 2023). The pressure for heavy preharvest antibiotic dependence is usually profit-driven and may arise from intensive farming operations or integrated farming systems, consequently exacerbating AMR spread and impacting consumer health (Adebowale et al., 2025).

In many low- and middle-income countries, including Nigeria, the motivations for heavy preharvest antibiotic use are multifactorial. Farmers in commercial poultry and swine operations often administer antibiotics prophylactically as an economic safeguard—protecting against disease-related losses in high-density production environments (Daodu et al., 2025). This tendency is further reinforced by the unrestricted over-the-counter availability of veterinary antimicrobials, which removes the barrier of professional veterinary oversight and enables self-prescription and sub-therapeutic dosing (Ndahi et al., 2023). Compounding these pressures are deficiencies in on-farm biosecurity, including inadequate sanitation protocols, insufficient vaccination programs, and poor waste management, which increase disease susceptibility and, in turn, drive further antibiotic dependence (Adebowale et al., 2025). Studies in Southwest Nigeria have previously documented high rates of antimicrobial use (AMU) on farms, with frequent application of tetracyclines, sulfonamides, and fluoroquinolones (Adebowale et al., 2025). This widespread, often sub-therapeutic use of preharvest antibiotics exerts significant selective pressure on bacterial populations, facilitating the development and persistence of multi-drug resistant (MDR) *Salmonella* in food-producing animals (Adebowale et al., 2025; Aworh et al., 2024; Ndahi et al., 2023). Several high-income countries, including the United States, China, Mexico, and the European Union, have implemented regulatory restrictions on preharvest antimicrobial use in livestock production systems, complemented by integrated monitoring programs (Zheng et al., 2025). This is exemplified by the Veterinary Feed Directive (VFD) and the National Antimicrobial Resistance Monitoring System (NARMS) which govern medically important antibiotic use in food animals in the United States, the European Union Regulation (EU) 2019/6, which prohibits the use of antibiotics for growth promotion and mandates veterinary prescription for therapeutic use, and the Action Plan on Antibiotic Resistance, phasing out non-therapeutic use across livestock systems in China (Sarkar and Okafor, 2024). In contrast, low- and middle-income countries (LMICs) have implemented limited initiatives to monitor preharvest antimicrobial use in food-producing animals, precluding a comprehensive assessment of their contribution to AMR propagation (Ikhimiukor and Okeke, 2023).

Lagos, a major commercial hub not only in Southwestern Nigeria but also in the West African subregion, hosts a dense network of poultry and swine farms that supply animal protein to millions of consumers (NEA, 2020). The intensification of these production systems has been accompanied by increased reliance on antibiotics, frequently administered without veterinary supervision (Adesehinwa et al., 2024). Previous studies in parts of Nigeria have documented the presence of multidrug-resistant *Salmonella* in livestock and retail meat products (Igbinosa et al., 2022; Aworh et al., 2024; Dagah et al., 2024), yet the specific contribution of preharvest antibiotic practices to resistance patterns remains poorly understood. This gap is especially critical in Lagos, where high population density, complex food supply chains, and close human–animal interactions may amplify the public health implications of AMR (Nazir et al., 2025). Although current investigations in Nigeria have largely focused on the prevalence of pathogens and the characterization of AMR in these pathogens, research seldom provides a comprehensive assessment of preharvest drivers of AMR during animal production. While phenotypic resistance data exist, molecular determinants driving microbial virulence and multidrug resistance remain poorly characterized. Additionally, studies elaborating the interplay between preharvest antibiotic use, farm biosecurity, and antimicrobial resistance have received limited attention, hampering risk assessment for zoonotic pathogen transmission (Odey et al., 2023). Previous investigations conducted across regions of Nigeria have documented the occurrence of multidrug-resistant *Salmonella* in livestock and retail meat products. Notably, Igbinosa et al. (2022) characterized the antimicrobial resistance profiles and genetic determinants of *Salmonella* enterica recovered from retail poultry meats in Benin City, while Aworh et al. (2024) reported the isolation of rare non-typhoidal *Salmonella* serovars from humans, beef cattle, and abattoir environments across Nigeria. Notwithstanding these contributions, there is a need for more specific, data-driven evidence linking preharvest antimicrobial use patterns directly to *Salmonella* resistance profiles.

Understanding how preharvest antibiotic use shapes resistance in *Salmonella* is essential for designing targeted One Health interventions, informing policy, and safeguarding both animal and human health. This study investigates the relationship between preharvest antibiotic use and the antibiotic resistance profiles of *Salmonella* isolated from commercial poultry and swine farms in Lagos, Southwestern Nigeria. Specifically, we aimed to: (i) determine the prevalence of *Salmonella* across farm types; (ii) characterize phenotypic and molecular AMR profiles; (iii) assess farm management and antibiotic use practices; and (iv) evaluate correlations between antibiotic use and resistance patterns to inform antimicrobial stewardship in livestock production.

## Materials and methods

### Study area

The study was conducted in Lagos State, Nigeria, a major commercial hub and the most populous state in West Africa, with intensive food animal production systems that serve both local and regional markets (Adesehinwa et al., 2024). Lagos State’s strategic location, diverse agricultural landscape, and significant role in Nigeria’s food supply chain make it an ideal setting for investigating *Salmonella* prevalence and AMR patterns with broader regional implications (Daramola and Makinde, 2024).

### Study design, sample size determination, and sample collection

A cross-sectional study was conducted between June 2023 and January 2024 across commercial poultry and pig farms in Lagos State, Nigeria. The study employed administration of structured questionnaires to farm owners and managers to assess farm management practices and antimicrobial use patterns, alongside the collection of fecal samples from layers, broilers, and pigs for microbiological and molecular analysis. Sample size was determined using standard epidemiological methods for prevalence studies (Thrusfield, 2008), with an assumed prevalence of 50% to maximize sample size in the absence of baseline data, 95 confidence level, and 5% precision. This yielded a minimum of 384 samples per animal species. To account for potential losses during collection and processing, and to ensure comprehensive geographic coverage, the target was increased to 400 samples per animal species across all three senatorial districts of Lagos State, totaling 3,600 fecal samples (1,200 samples each collected from layers, broilers, and pigs). Fecal samples were methodically collected from layers, broilers, and pigs on randomly selected commercial farms across the three senatorial districts of Lagos State, Nigeria. Samples were collected aseptically using sterile screw-cap containers, assigned unique identifiers, and transported under cold-chain conditions to preserve sample integrity before laboratory analysis (Waktole et al., 2024).

### Isolation and identification of *Salmonella*

The isolation of *Salmonella* from fecal samples was performed in accordance with the International Organization for Standardization protocol ISO 6579–1:2017 (ISO, 2017). Non-selective enrichment was performed by aseptically transferring 1g of each sample to 10 mL Buffered Peptone Water (BPW) (Neogen^®^ Culture Media United Kingdom). The samples were homogenized by gentle vortexing (2 min) and incubated (37°C, 18–24 h), with turbidity confirming growth (Mukuna et al., 2023). Selective enrichment employed 0.1mL of the non-selective pre-enrichment broth inoculated into 10 mL Rappaport-Vassiliadis Soy broth (RVS) (TM Culture Media, India) and 1 mL into 10 mL Tetrathionate broth (TTB) (TM Culture Media, India), incubated at 42 and 37°C, respectively, for 18–24 h. Selective enrichment cultures (0.01 mL) were streaked onto Xylose Lysine Deoxycholate agar (Neogen^®^ Culture Media United Kingdom) and incubated (37°C, 18–24 h). Presumptive *Salmonella* colonies (red-pink halos with black centers) were subcultured on nutrient agar (37°C, 18–24 h). The isolates were cryopreserved at −80°C in 30% sterile glycerol for subsequent molecular analysis (ISO, 2017). The obtained pure isolates were subjected to Gram staining and a comprehensive panel of biochemical assays. The analytical scheme incorporated catalase (Ct), coagulase (Cg), urease (URE), indole (I), methyl-red (M), Voges-Proskauer (VP), citrate utilization (CIT), and triple sugar iron (TSI) tests. All procedures were conducted in accordance with standardized protocols as described by Cheesbrough (2006).

### Phenotypic antimicrobial susceptibility testing

Antimicrobial susceptibility profiles were determined using the Kirby-Bauer disc diffusion method in strict accordance with Clinical and Laboratory Standards Institute (CLSI) guidelines (CLSI, 2024). The antimicrobial panel was selected to represent major antibiotic classes used in both veterinary and human medicine, including fluoroquinolones (ciprofloxacin 5 μg, nalidixic acid 30 μg, enrofloxacin 5 μg), aminoglycosides (gentamicin 5 μg, streptomycin 25 μg), macrolides (erythromycin 10 μg), phenicols (chloramphenicol 30 μg), tetracyclines (tetracycline 10 μg), β-lactams (ampicillin 10 μg), and folate pathway inhibitors (trimethoprim-sulfamethoxazole 25 μg). Bacterial suspensions were prepared to a 0.5 McFarland turbidity standard using sterile normal saline to ensure a standardized inoculum density. Sterile swabs were used to create uniform bacterial lawns on Mueller-Hinton agar plates, with antimicrobial discs (Oxoid Ltd., Basingstoke, United Kingdom) applied aseptically. Plates were incubated at 35°C for 18–24 h under aerobic conditions. Inhibition zone diameters were measured to the nearest millimeter using a ruler and interpreted according to CLSI breakpoints to classify isolates as susceptible, intermediate, or resistant. The Multiple Antimicrobial Resistance (MAR) index was calculated for each isolate as the ratio of the number of antibiotics to which the isolate showed resistance to the total number of antibiotics tested (CLSI, 2022). This index provides a quantitative measure of multidrug resistance, with values > 0.2 indicating isolates from high-risk sources with significant antimicrobial exposure.

### Molecular identification of salmonella

Genomic DNA extraction from *Salmonella* cultures incubated overnight at 37°C was accomplished using the Nucleospin Microbial DNA kit (Duren, Germany) according to the manufacturer’s protocol. Quantification and quality assessment of extracted DNA were conducted using a NanoDrop Lite spectrophotometer (Thermo Scientific, Waltham, MA, United States) adhering to established protocols (Mukuna et al., 2023). Verification of *Salmonella* isolates was achieved through amplification of the genus-specific *invA* gene, utilizing primer sequences detailed in Supplementary Table 1. The PCR reaction was prepared in a 10 μL total volume comprising 0.2 μL of each primer (forward and reverse), 5 μL TaqMan master mix (containing Tris-HCl pH 8.5, (NH_4_)_2_SO_4_, 3 mM MgCl_2_, 0.2% Tween 20, 0.4 mM dNTPs, Ampliqon Taq DNA polymerase, inert red dye, and stabilizer), 1.5 μL template DNA, and 3.1 μL molecular-grade water; nuclease-free water served as the negative control. The thermal cycling protocol (VWR, Germany) involved initial denaturation at 95°C for 5 min, succeeded by 34 amplification cycles (95°C for 30 s, 58°C for 30 s, 72°C for 1 min), and completed with a final extension step at 72°C for 5 min. Amplified products were resolved via 1.5% agarose gel electrophoresis at 70 V for 60 min, generating amplicons corresponding to target genes (Zishiri et al., 2016). Gel visualization was performed using the VWR Imager2 gel documentation system (Germany).

### Detection of antimicrobial resistance and virulence genes

Antimicrobial resistance genes associated with major antibiotic classes were detected using primers and PCR conditions specified in Supplementary Table 1. Target genes included *tetA* (tetracycline resistance), *ant*(3”)-*Ia* (aminoglycoside resistance), *sul1* (sulfonamide resistance), *blaCTX-M* (β-lactam resistance), and *qnr* genes (quinolone resistance). PCR reactions were performed in 10 μL volumes following established protocols with annealing temperatures optimized for each gene target (Herrera-Sánchez et al., 2021). *Salmonella* virulence genes including *iroB*, *spiC*, *orfL*, and *pipD* were amplified using primers from Supplementary Table 1 with optimized thermal cycling conditions (Mthembu et al., 2019; Zishiri et al., 2016). All amplified products were analyzed by 1.5% agarose gel electrophoresis (70V, 60 min) and visualized using the VWR gel documentation system.

### Farm management and antibiotic use assessment

A structured questionnaire was developed and administered to farm managers/owners to assess farm management practices, antimicrobial use patterns, and biosecurity measures. The questionnaire was designed in English and modified based on previous work in antimicrobial use surveys (Adebowale et al., 2020; Gibson et al., 2004). Two specialists in biosecurity and antimicrobial use/resistance examined and authenticated the questionnaire content. Pilot testing involving two farmers from non-study locations was undertaken to assess the instrument’s relevance, comprehensiveness, and interpretability. Refinements identified through participant feedback and expert recommendations were implemented before widespread deployment. Between July and November 2023, the research personnel administered the questionnaire during on-site sample collection visits. The instrument featured predominantly structured questions to facilitate efficient data management and response accuracy, complemented by unstructured items when appropriate. The survey instrument was organized into distinct segments capturing details regarding: Farm demographics and characteristics (farm type, production system, flock/herd size); Biosecurity and management practices (presence of biosecurity protocols, waste management, vaccination programs, visitor control, disease prevention measures); Antimicrobial use patterns (types of antimicrobials used, purposes of use—treatment, prophylaxis, growth promotion); Drug prescription practices (self-medication versus veterinary consultation) (Supplementary File 2). Participation was voluntary, and verbal informed consent was obtained from all participants after a detailed explanation of the study objectives and data collection procedures. Farms were required to have active production operations at the time of sampling and maintain accessible records of flock/herd management practices. No specific exclusion criteria were applied; however, farms that declined participation or were unable to provide the required fecal samples due to operational constraints were not included in the study. All data were handled confidentially and anonymously in accordance with standard research practices.

### Statistical analysis

Data were analyzed using descriptive and inferential statistical methods. Questionnaire data on antimicrobial use patterns were integrated with laboratory data on AMR profiles. Summary statistics included frequency distributions, means, and measures of dispersion. Inferential analyses included ANOVA, Cramer’s V test, and Pearson’s correlation coefficient, performed using GraphPad Prism^1^ and Python via Jupyter Notebook,^2^ with significance set at *p* < 0.05.

## Results

### Salmonella prevalence in food animals

From 3,600 fecal samples collected across layers, broilers, and pigs in three senatorial zones of Lagos State, Nigeria, 54 isolates were confirmed as *Salmonella*, representing an overall prevalence of 1.5% (Table 1). We found significant differences in *Salmonella* prevalence across animal types, with pigs demonstrating significantly higher prevalence than both layers (*p* < 0.0001) and broilers (*p* = 0.0055). Broilers exhibited significantly higher prevalence than layers (*p* < 0.0001), revealing a clear hierarchical pattern of infection: pigs > broilers > layers. Our analysis revealed marked regional variation in *Salmonella* distribution. Lagos West demonstrated the highest prevalence at 2.6% (31/1,200), significantly exceeding both Lagos East (0.92%, 11/1,200; *p* < 0.0001) and Lagos Central (1.0%, 12/1,200; *p* < 0.0001). No significant difference was observed between Lagos East and Lagos Central (*p* > 0.9999), suggesting comparable infection levels in these zones despite the markedly elevated prevalence in Lagos West (Table 1).

**TABLE 1:** Occurrence of *Salmonella* from food animals in Lagos State.

S/N	Animal type	Sample size	Senatorial zone			Total	Adjusted *P*-value: multiple comparison
			Lagos west (W)	Lagos east (E)	Lagos central (C)		
1	Layers (L)	1,200	3 (0.25%)	0 (0.00%)	5 (0.42%)	8 (0.67%)	L vs. B
							< 0.0001
2	Broilers (B)	1,200	10 (0.83%)	7(0.58%)	3 (0.25%)	20 (1.67)	B vs. P
							0.0055
3	Pigs (P)	1,200	18 (1.50%)	4 (0.33%)	4 (0.33%)	26 (2.2%)	L vs. P
							< 0.0001
	Total	3,600	31 (2.6%)	11 (0.92%)	12 (1%)	54(1.5%)	
	Adjusted *P*-value: multiple comparison		W vs. E < 0.0001	E vs. C > 0.9999	W vs. C < 0.0001		

*P*-value for animal type by senatorial zone < 0.001. *p* < 0.05 are statistically significant.

### Antimicrobial resistance profiles and patterns

Antimicrobial susceptibility testing of *Salmonella* isolates against nine commonly used antibiotics revealed widespread resistance across all the animal types (Table 2). The nine antibiotics tested represented six antibiotic classes: fluoroquinolones (ciprofloxacin, nalidixic acid), aminoglycosides (gentamicin, streptomycin), macrolides (erythromycin), phenicols (chloramphenicol), tetracyclines (tetracycline), β-lactams (ampicillin), and folate pathway inhibitors (trimethoprim-sulfamethoxazole). In this study, an isolate was classified as multidrug-resistant (MDR) when it demonstrated resistance to at least one antibiotic within each of three or more distinct antimicrobial classes, consistent with the internationally recognized criteria established by Magiorakos et al. (2012). Detailed antimicrobial resistance profiles for individual isolates are presented in Table 3 for layers, broilers, and pigs, respectively. Layer-derived isolates (*n* = 8) exhibited high resistance rates to erythromycin (100%, 8/8), nalidixic acid (75%, 6/8), ampicillin (75%, 6/8), and tetracycline (75%, 6/8), while demonstrating the highest susceptibility to sulfamethoxazole-trimethoprim (75%, 6/8) and chloramphenicol (62.5%, 5/8). Broiler-derived isolates (*n* = 20) showed high resistance to tetracycline (90.0%, 18/20), erythromycin (80.0%, 16/20), sulfamethoxazole-trimethoprim (80.0%, 16/20), nalidixic acid (80.0%, 16/20), and ampicillin (85.0%, 17/20), with gentamicin displaying the highest efficacy (95% susceptibility, 19/20). Pig-derived isolates (*n* = 26) demonstrated the most concerning resistance profile, with universal resistance to erythromycin (100%, 26/26) and near-universal resistance to tetracycline (92.3%, 24/26), while retaining notable susceptibility to gentamicin (84.6%, 22/26) and ciprofloxacin (57.7%, 15/26). Multiple Antibiotic Resistance Index (MARI) values ranged from 0.2 to 0.9 across all isolates, indicating extensive multidrug resistance. Notably, the highest MARI values of 0.9 were observed in layer isolate L175-S and broiler isolate B654-S, both of which were resistant to eight of nine tested antibiotics. Pig-derived isolates exhibited maximum resistance to seven antibiotics (MARI = 0.8), with several isolates displaying this resistance pattern (Table 3).

**TABLE 2:** Antimicrobial susceptibility profile of *Salmonella* from layers, broilers, and pigs.

Antibiotics	Layers		Broilers			Pigs
	Resistant (%)	Susceptible (%)	Resistant (%)	Susceptible (%)	Resistant (%)	Susceptible (%)
TE	6 (75.0)	2 (25.0)	18 (90.0)	0 (0.0)	24(92.3)	0(0.0)
E	8 (100.0)	0 (0.0)	16 (80.0)	4 (20.0)	26(100.0)	0(0.0)
C	1 (12.5)	5 (62.5)	7 (35.0)	3 (15.0)	3(11.5)	11(42.3)
SXT	2 (25.0)	6 (75.0)	16 (80.0)	4 (20.0)	12(46.2)	11(42.3)
NA	6 (75)	0 (0.0)	16 (80.0)	0 (0.0)	12(46.2)	4(15.3)
S	2 (25.0)	3 (37.5)	13 (65.0)	5 (25.0)	10(38.5)	11(42.3)
CIP	4 (50.0)	0 (0.0)	13 (65.0)	1 (5.0)	4(15.4)	15(57.7)
AMP	6 (75.0)	2 (25.0)	17 (85.0)	2 (10.0)	11(42.3)	9(34.6)
CN	2 (25.0)	3 (37.5)	0 (0.0)	19 (95.0)	1(3.9)	22(84.6)

TE, tetracycline; E, erythromycin; C, chloramphenicol; SXT, sulfamethoxazole-trimethoprim; NA, nalidixic acid; S, streptomycin; AMP, ampicillin; CN, gentamicin; CIP, ciprofloxacin.

**TABLE 3:** Antimicrobial resistance phenotypic and genotypic patterns of *Salmonella* from layers broilers and pigs.

SN	Source	Isolate ID	No. of resistance	MARI	AMR phenotype	AMR genotype
Layers						
1		L946-S	2	0.2	TE, E	Sul1, ant(3″)-la, tetA
2		L865-S	2	0.2	E, AMP	Sul1, ant(3″)-la, tetA
3		L826-S	4	0.4	TE, E, NA, CIP	ant(3″)-la, tetA
4		L848-S	4	0.4	TE, E, NA, AMP	Sul1, ant(3″)-la, tetA
5		L191-S	5	0.6	TE, E, NA, CIP, CN	Sul1, ant(3″)-la, tetA
6		L875-S	6	0.7	TE, E, NA, S, CIP, AMP	qnrB, ant(3″)-la, tetA
7		L945-S	6	0.7	TE, E, SXT, NA, S, AMP	qnrB, Sul1, ant(3″)-la
8		L175-S	8	0.9	TE, E, C, SXT, NA, CIP, AMP, CN	qnrB, Sul1, ant(3″)-la, tetA
Broilers						
1		B362-S	2	0.2	TE, NA	qnrS, Sul1, ant(3″)-la, tetA
2		B347-S	4	0.4	SXT, NA, CIP, AMP	qnrB, qnrS, ant(3″)-la
3		B456-S	5	0.6	TE, E, SXT, S, AMP	Sul1, ant(3″)-la
4		B302-S	5	0.6	TE, E, SXT, S, AMP	qnrS, ant(3″)-la, tetA
5		B104-S	5	0.6	TE, E, NA, CIP, AMP	qnrS, Sul1, ant(3″)-la
6		B376-S	5	0.6	TE, SXT, NA, CIP, AMP	qnrB, qnrS, Sul1, ant(3″)-la, tetA
7		B309-S	6	0.7	TE, E, C, NA, S, CIP	qnrS, ant(3″)-la, tetA
8		B321-S	6	0.7	TE, E, C, S, CIP, AMP	Sul1, ant(3″)-la
9		B192-S	6	0.7	E, C, SXT, NA, S, AMP,	qnrS, ant(3″)-la, tetA
10		B477-S	6	0.7	TE, E, SXT, S, CIP, AMP	qnrS, Sul1, ant(3″)-la, tetA
11		B638-S	6	0.7	TE, E, SXT, NA, S, AMP	qnrS, Sul1, ant(3″)-la, tetA
12		B488-S	6	0.7	TE, E, SXT, NA, S, AMP	qnrB, qnrS, Sul1, ant(3″)-la, tetA
13		B784-S	6	0.7	TE, E, SXT, NA, CIP, AMP	qnrB, qnrS, Sul1, ant(3″)-la, tetA
14		B827-S	6	0.7	TE, E, SXT, NA, CIP, AMP	qnrS, Sul1, ant(3″)-la, tetA
15		B345-S	6	0.7	TE, C, SXT, NA, CIP, AMP	qnrB, qnrS, Sul1, ant(3″)-la
16		B618-S	7	0.8	TE, E, C, SXT, NA, S, AMP	qnrB, qnrS, Sul1
17		B351-S	7	0.8	TE, E, C, SXT, NA, CIP, AMP	qnrB, qnrS, Sul1, ant(3″)-la, tetA
18		B729-S	7	0.8	TE, E, SXT, NA, S, CIP, AMP	Sul1, ant(3″)-la, tetA
19		B643-S	7	0.8	TE, E, SXT, NA, S, CIP, AMP	qnrB, qnrS, Sul1, ant(3″)-la, tetA
20		B654-S	8	0.9	TE, E, C, SXT, NA, S, CIP, AMP	Sul1, ant(3″)-la, tetA
Pigs						
1		P253-S	2	0.2	TE, E	qnrB, ant(3″)-la, tetA
2		P49-S	2	0.2	TE, E	Sul1, ant(3″)-la, tetA
3		P66-S	2	0.2	TE, E	qnrB, qnrS, Sul1, ant(3″)-la, tetA
4		P37-S	2	0.2	TE, E	qnrB, qnrS, Sul1, ant(3″)-la, tetA
5		P109-S	2	0.2	TE, E	qnrS, Sul1, ant(3″)-la, tetA
6		P34-S	2	0.2	TE, E	qnrB, ant(3″)-la, tetA
7		P65-S	2	0.2	TE, E	qnrB, qnrS, Sul1, ant(3″)-la, tetA
8		P38-S	2	0.2	TE, E	qnrS, ant(3″)-la, tetA
9		P71-S	2	0.2	TE, E	qnrB, ant(3″)-la, tetA
10		P283-S	2	0.2	TE, E	qnrB, qnrS, Sul1, ant(3″)-la, tetA
11		P222-S	3	0.3	TE, E, SXT	qnrS, Sul1, tetA
12		P353-S	4	0.4	TE, E, NA, S	qnrB, Sul1, tetA
13		P60-S	4	0.4	TE, E, NA, S	qnrB, Sul1, ant(3″)-la, tetA
14		P573-S	4	0.4	E, NA, S, AMP	qnrB, Sul1, tetA
15		P399-S	4	0.4	E, SXT, S, AMP	qnrB, tetA
16		P75-S	4	0.4	TE, E, SXT, AMP	qnrS, Sul1, ant(3″)-la, tetA
17		P76-S	5	0.6	TE, E, C, SXT, NA	Sul1, ant(3″)-la, tetA
18		P117-S	5	0.6	TE, E, SXT, S, AMP	qnrS, Sul1, ant(3″)-la, tetA
19		P33-S	5	0.6	TE, E, SXT, NA, CIP	qnrB, qnrS, ant(3″)-la, tetA
20		P414-S	5	0.6	TE, E, SXT, NA, AMP	qnrB, Sul1, ant(3″)-la, tetA
21		P274-S	6	0.7	TE, E, SXT, NA, S, AMP	qnrS, Sul1, ant(3″)-la, tetA
22		P498-S	6	0.7	TE, E, SXT, NA, S, AMP	Sul1, ant(3″)-la, tetA
23		P505-S	7	0.8	TE, E, C, SXT, NA, S, AMP	qnrB, Sul1, ant(3″)-la, tetA
24		P389-S	7	0.8	TE, E, C, NA, S, CIP, AMP	tetA
25		P393-S	7	0.8	TE, E, NA, S, CIP, AMP, CN	qnrB, qnrS, ant(3″)-la, tetA
26		P126-S	7	0.8	TE, E, C, SXT, NA, CIP, AMP	qnrB, qnrS, ant(3″)-la, tetA

TE, tetracycline; E, erythromycin; C, chloramphenicol; SXT, sulfamethoxazole-trimethoprim; NA, nalidixic acid; S, streptomycin; AMP, ampicillin; CN, gentamicin; CIP, ciprofloxacin.

### Distribution of antimicrobial resistance genes

**FIGURE 1:**
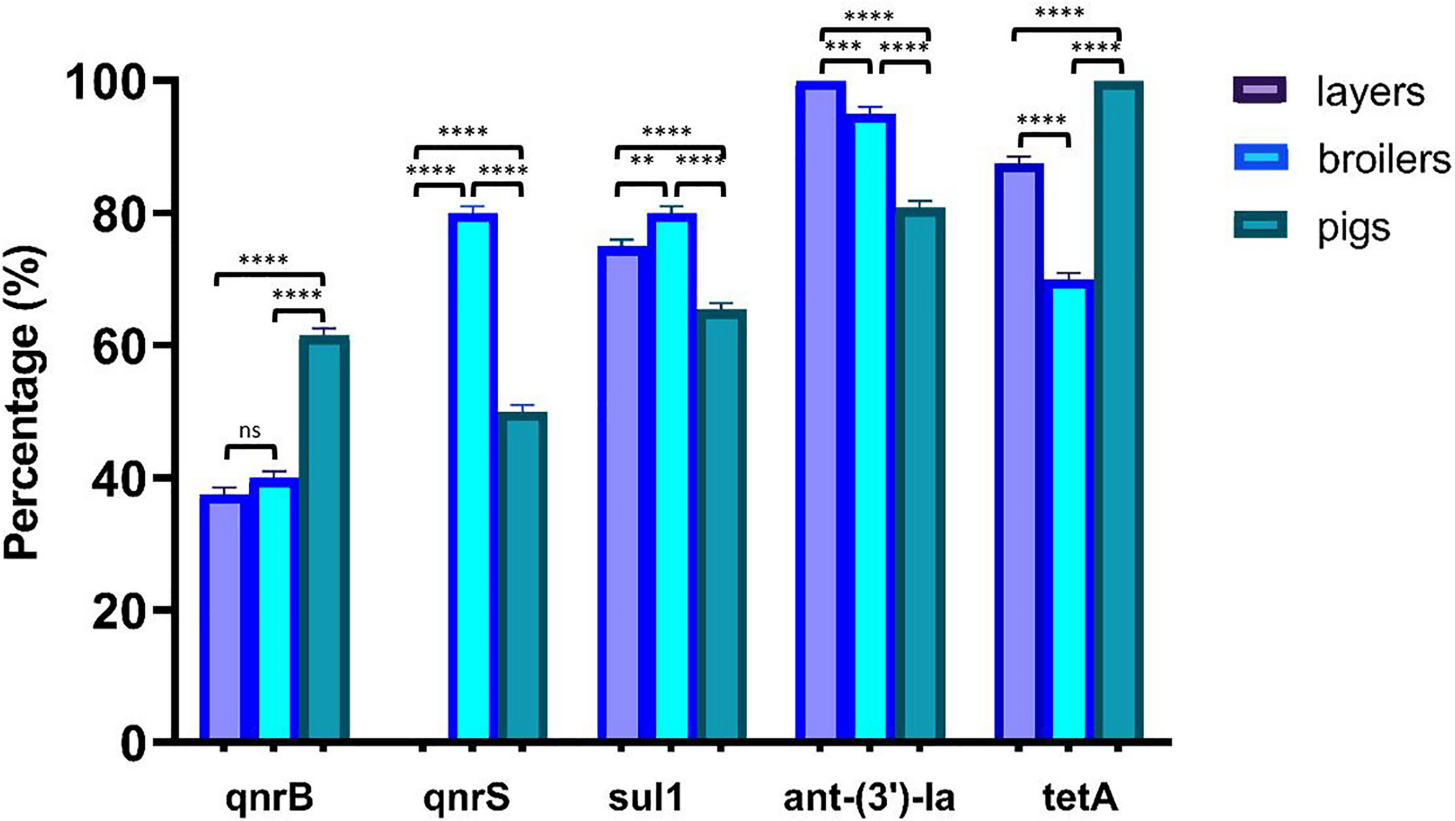
Distribution of AMR genes across *Salmonella* isolates from layers, broilers, and pigs. *P*-value significance: ****p < 0.0001; ***p < 0.001; **p < 0.01; *p < 0.05 ns (not significant) *p* > 0.05.

Molecular characterization of antimicrobial resistance genes revealed patterns that closely aligned with observed phenotypic resistance profiles (Figure 1). The tetracycline resistance gene *tetA* demonstrated near-universal distribution, present in 100% of isolates from layers and pigs, and 70% of broiler-derived isolates, strongly correlating with the high tetracycline resistance rates observed phenotypically. The aminoglycoside resistance gene *ant* (3”)*-Ia* showed complete presence (100%) in all layer-derived *Salmonella* isolates, 95% in broiler-derived isolates, and 80.8% in pig-derived isolates. The sulfamethoxazole resistance gene *Sul1* was detected in 75% of layer-derived isolates, 80% of broiler-derived isolates, and 65.4% of pig-derived isolates.

Quinolone resistance genes exhibited distinct host-specific distribution patterns. The *qnrB* gene was present in 37.5% of layer-derived isolates, 40% of broiler-derived isolates, and 61.5% of pig-derived isolates. The *qnrS* gene demonstrated marked variation, absent in layer-derived isolates (0%) but present in 80% of broiler-derived isolates and 50% of pig-derived isolates. The *qnrA* and *qnrC* genes were not detected across any animal species (Table 4). Despite observed ampicillin resistance, the β-lactamase gene *bla*CTX-M was absent in all isolates, suggesting alternative resistance mechanisms such as other β-lactamase variants or efflux pumps.

**TABLE 4:** Distribution of AMR genes across *Salmonella* isolates from layers, broilers, and pigs.

Sample type	No. of isolates	AMR genes							
		*qnrA*	*qnrB*	*qnrC*	*qnrS*	*sul1*	*bla_CTX-M*	*ant(3″)-la*	*tetA*
Layers	8	0	3	0	0	6	0	8	7
Broilers	20	0	8	0	16	16	0	19	14
Pigs	26	0	16	0	13	17	0	21	26
Total	54	0	27	0	29	39	0	48	47

**FIGURE 2:**
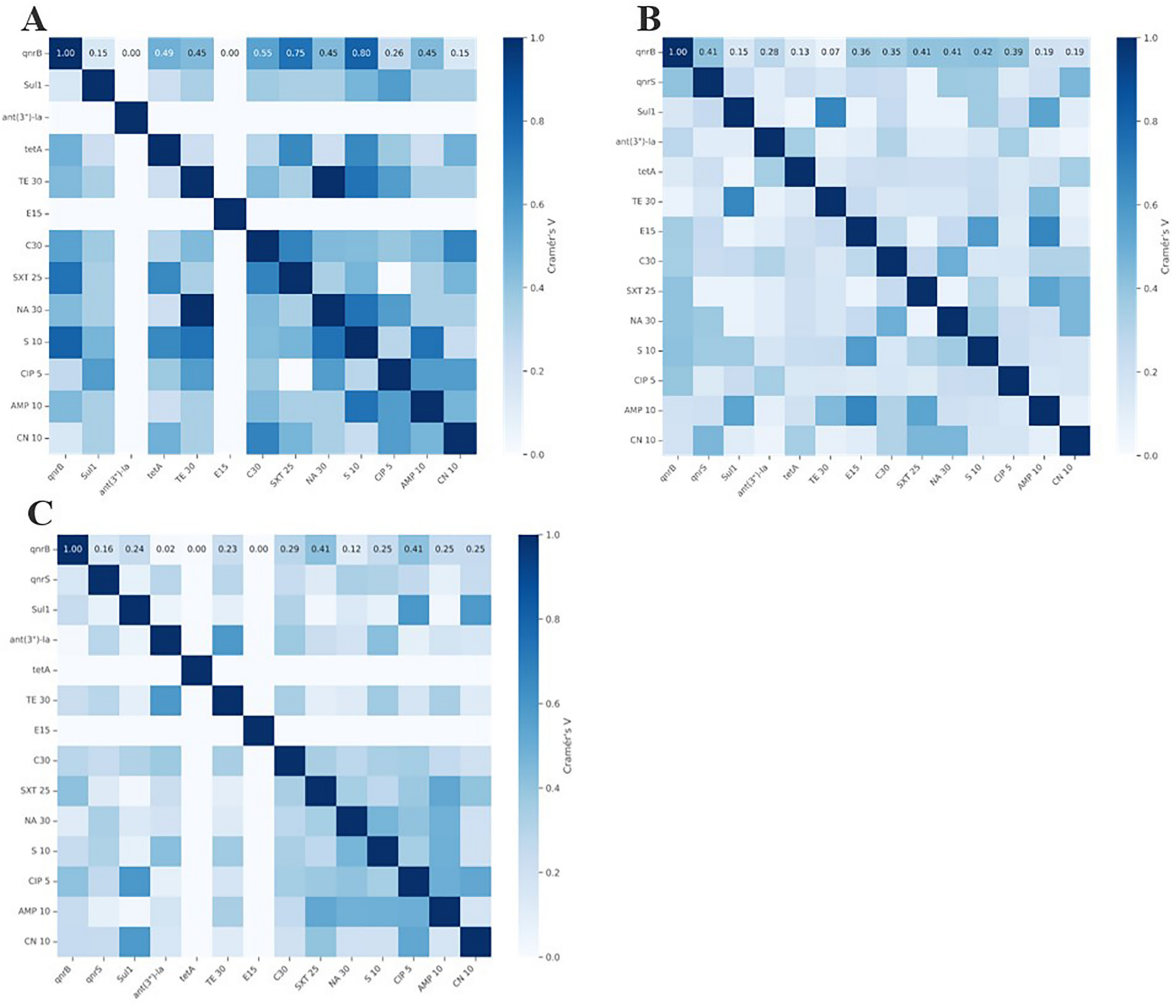
Association heatmap of AMR of genes and Phenotypes in *Salmonella* across the different sources. **(A)** Layers, **(B)** Broilers, **(C)** Pigs.

Correlation analysis between phenotypic resistance and genotypic markers revealed moderate to strong positive associations across the antimicrobial classes. Specifically, tetracycline phenotype-genotype association demonstrated moderate positive association χ^2^ = 0.38 (*p* = 0.537, Cramer’s V = 0.22), fluoroquinolone (nalidixic acid) resistance showed strong positive association χ^2^ = 1.60 (*p* = 0.206, Cramer’s V = 0.45), and sulfonamide resistance exhibited moderate positive association χ^2^ = 0.89 (*p* = 0.346, Cramer’s V = 0.33) in layer-derived isolates, indicating reliable concordance between detected resistance genes and expressed phenotypes (Figure 2).

### Virulence gene profiles

**FIGURE 3:**
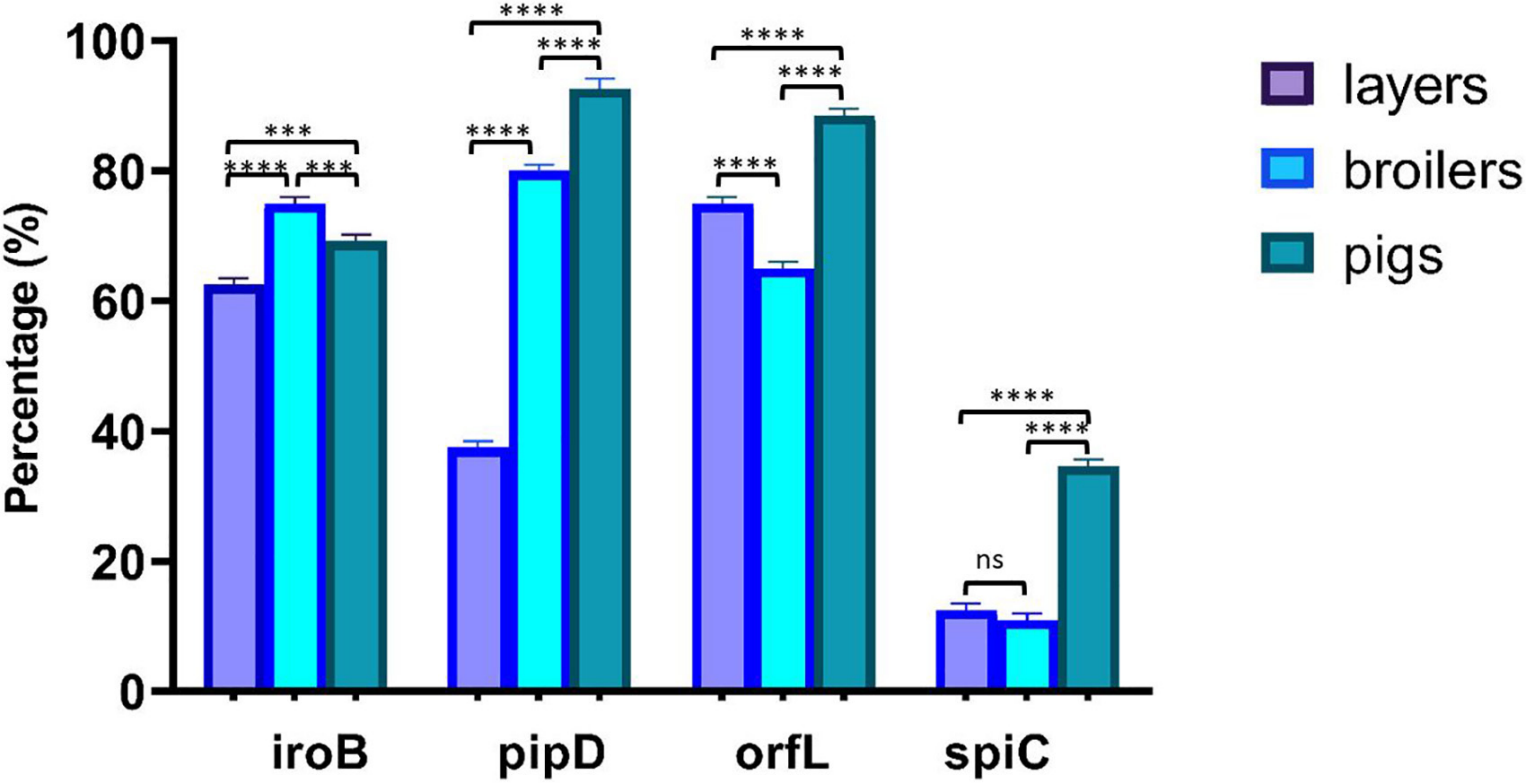
Distribution of virulence genes across *Salmonella* isolates from layers, broilers, and pigs. *P*-value significance: ****p < 0.0001; ***p < 0.001; **p < 0.01; *p < 0.05 ns (not significant) *p* > 0.05.

Virulence gene profiling revealed species-specific distribution patterns that reflect differences in pathogenic potential and host adaptation across animal sources (Figure 3 and Table 5). The iron acquisition gene *iroB*, critical for bacterial survival in host environments, showed variable distribution: 62.5% in layer-derived *Salmonella* isolates, 75% in broiler-derived isolates, and 69.2% in pig-derived isolates, indicating widespread but non-universal iron acquisition capabilities. Pathogenicity island genes demonstrated progressive increases from layers to pigs, suggesting enhanced virulence potential in pig-derived isolates. The *pipD* gene, associated with systemic infection capability, exhibited a clear gradient: 37.5% in layer-derived isolates, 80% in broiler-derived isolates, and 92.3% in pig-derived isolates. The *orfL* gene followed similar trends with prevalence rates of 75% in layer-derived isolates, 65% in broiler-derived isolates, and 88.5% in pig-derived isolates. In contrast, the type III secretion system gene *spiC* showed uniformly low prevalence across all species: 12.5% in layer-derived isolates, 10% in broiler-derived isolates, and 34.6% in pig-derived isolates, indicating limited diversity in this secretion system component among the isolates.

**TABLE 5:** Distribution of virulence genes across *Salmonella* isolates from layers, broilers, and pigs.

Sample type	No. of isolates	Virulence genes			
		*iroB*	*pipD*	*orfL*	*spiC*
Layers	8	5	3	6	1
Broilers	20	15	16	13	2
Pigs	26	18	24	23	9
Total	54	38	43	42	12

### Farm management practices and antimicrobial use patterns

**FIGURE 4:**
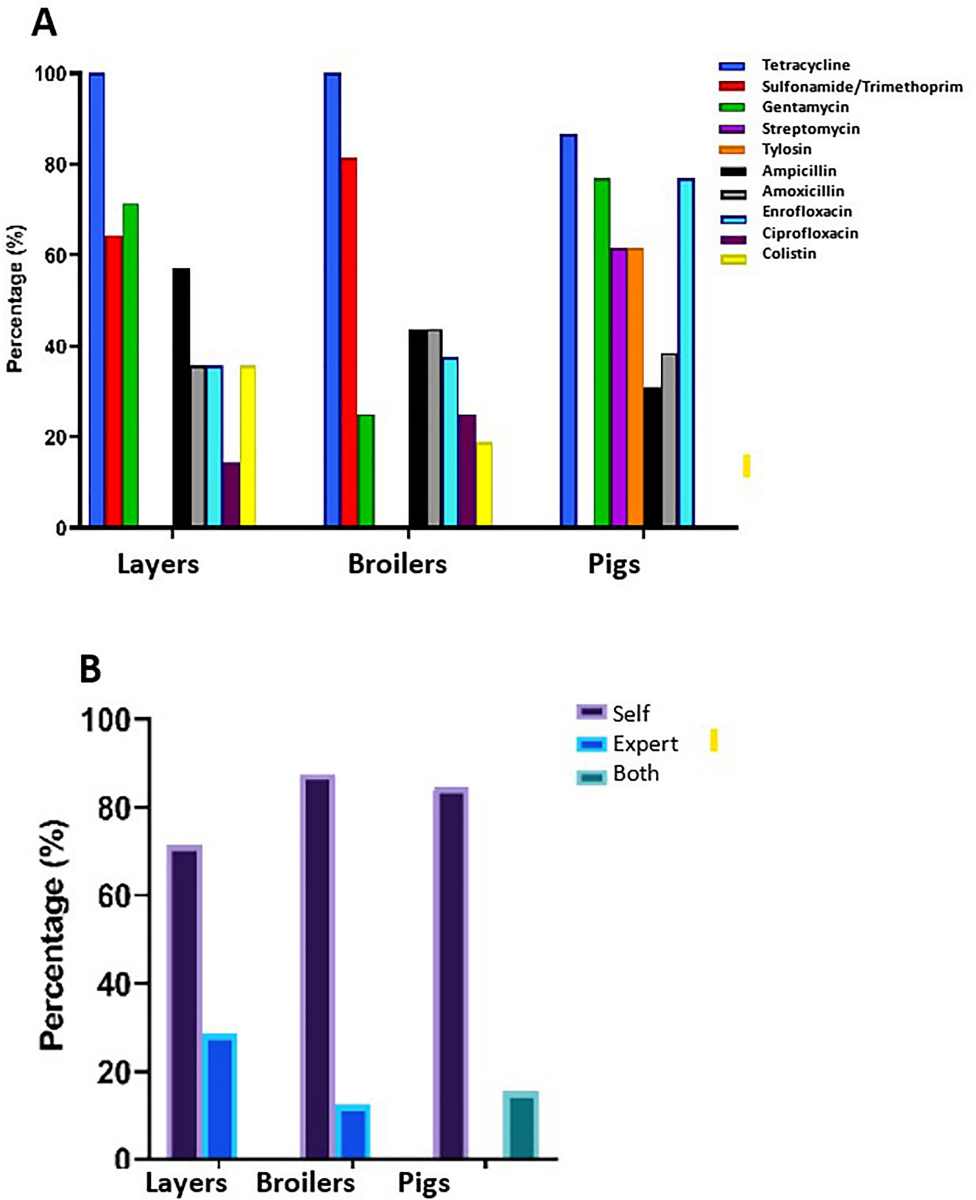
**(A)** Antimicrobial use by Farm type. **(B)** Prescription of antibiotics by farm type.

**FIGURE 5:**
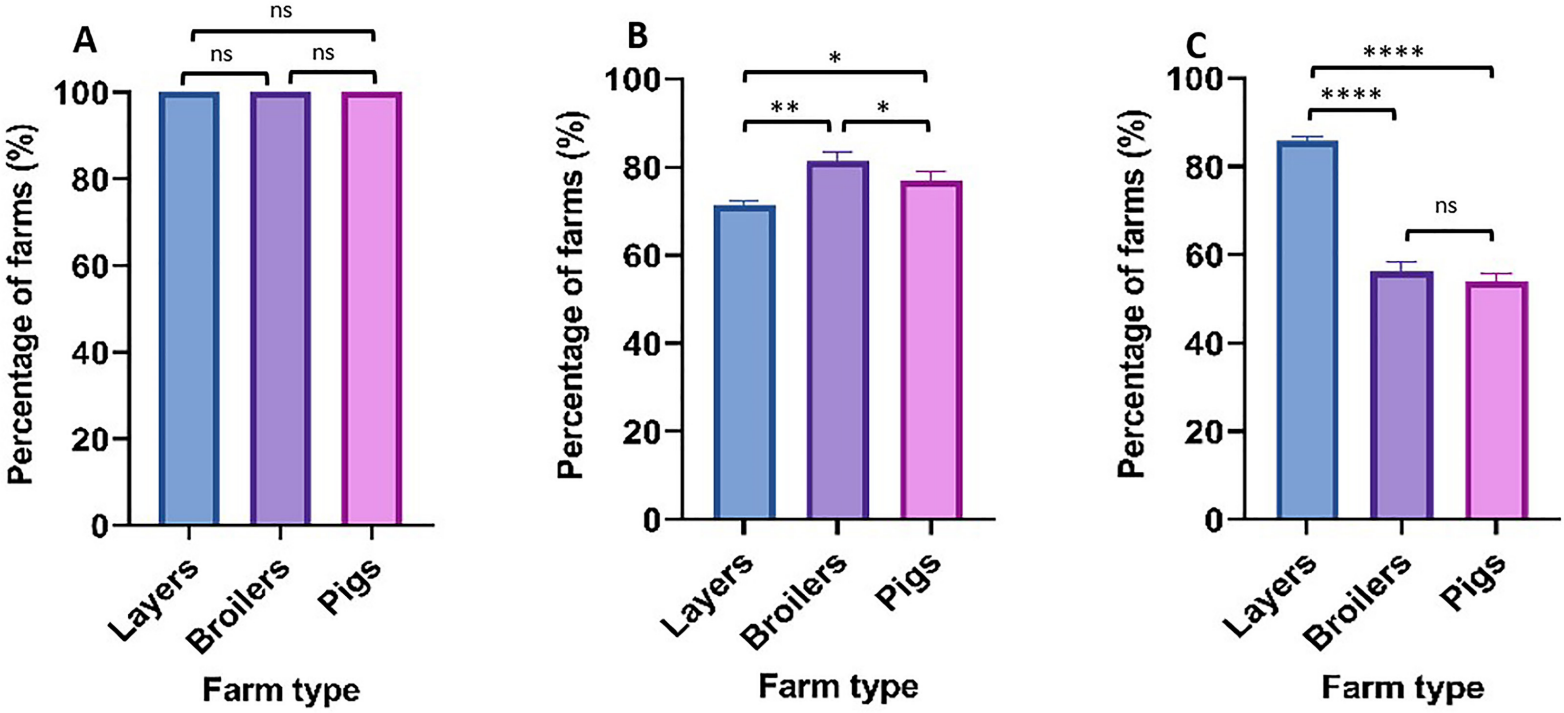
Antibiotic use on animals by farm type. **(A)** Treatment, **(B)** Prophylaxis, **(C)** Growth promotion. *P*-value significance: ****p < 0.0001; ***p < 0.001; **p < 0.01; *p < 0.05 ns (not significant) *p* > 0.05.

Comprehensive assessment of farm management practices revealed concerning antimicrobial use patterns across all production systems. All surveyed farms (100%) operated as conventional systems (Figure 4). Growth promotion practices showed substantial variation, with antimicrobials used for this purpose in 85.7% of layer farms, 56.3% of broiler farms, and 53.8% of pig farms. Therapeutic antimicrobial use for treatment of clinical disease was universal (100%) across all farm types, while prophylactic use demonstrated moderate variation: 71.4% in layer farms, 81.3% in broiler farms, and 71.4% in pig farms (Figure 5). Flock size correlated with antimicrobial use for growth promotion but showed no significant association with treatment or prophylactic use patterns (Supplementary Figure 5). Ten antibiotics were documented across surveyed farms for animal production purposes (Figure 4). Tetracycline emerged as the most frequently used antimicrobial, employed in 100% of layer farms, 86.6% of broiler farms, and 100% of pig farms. Sulfamethoxazole-trimethoprim ranked second in usage frequency (64.3% of layer farms, 81.3% of broiler farms, 0% of pig farms), followed by ampicillin (57.1% of layer farms, 43.8% of broiler farms, 30.8% of pig farms). Gentamicin use was prevalent in layer (71.4%) and pig (76.9%) farms but considerably less common in broiler operations (25%). Drug prescription practices revealed alarming self-medication trends, with the majority of farmers administering antimicrobials without veterinary consultation or supervision: 71.4% of layer farmers, 87.5% of broiler farmers, and 84.6% of pig farmers. Professional veterinary consultation was limited to 28.6% of layer farms and 12.5% of broiler farms, while 15.4% of pig farms reported using both veterinary consultation and self-medication practices (Figure 4).

**FIGURE 6:**
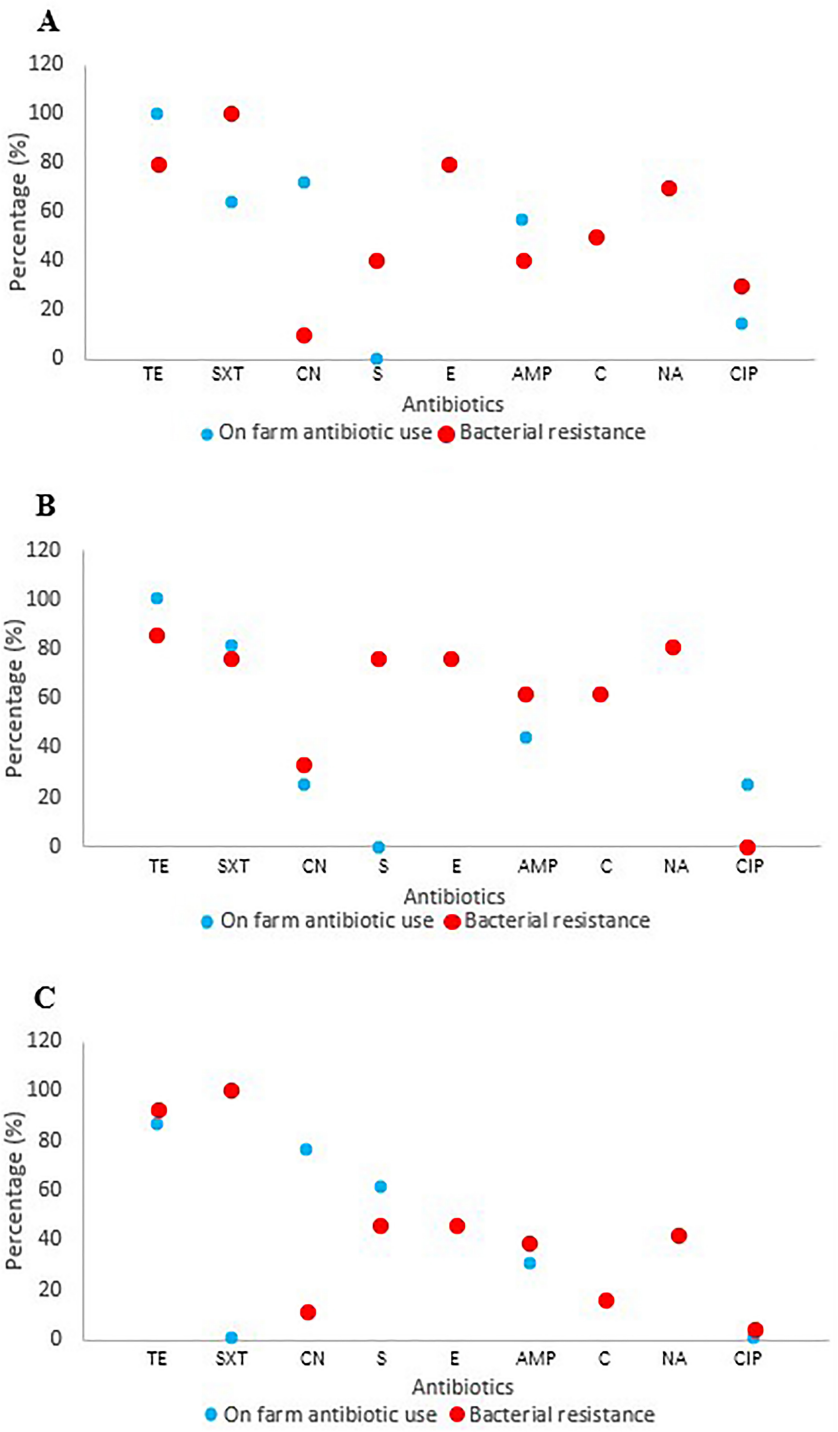
Correlation of on-farm antibiotic use and antibiotic resistance in *Salmonella* isolates. **(A)** Layer farms (*r* = 0.400, *p* = 0.432), **(B)** Broiler farms (*r* = 0.487, *p* = 0.327), **(C)** Pig farms (Pigs: *r* = 0.058, *p* = 0.913). *r* < 0.5 imply weak correlation; *p* > 0.05 are not significant. TE, tetracycline; E, erythromycin; C, chloramphenicol; SXT, sulfamethoxazole-trimethoprim; NA, nalidixic acid; S, streptomycin; AMP, ampicillin; CN, gentamicin; CIP, ciprofloxacin.

**FIGURE 7:**
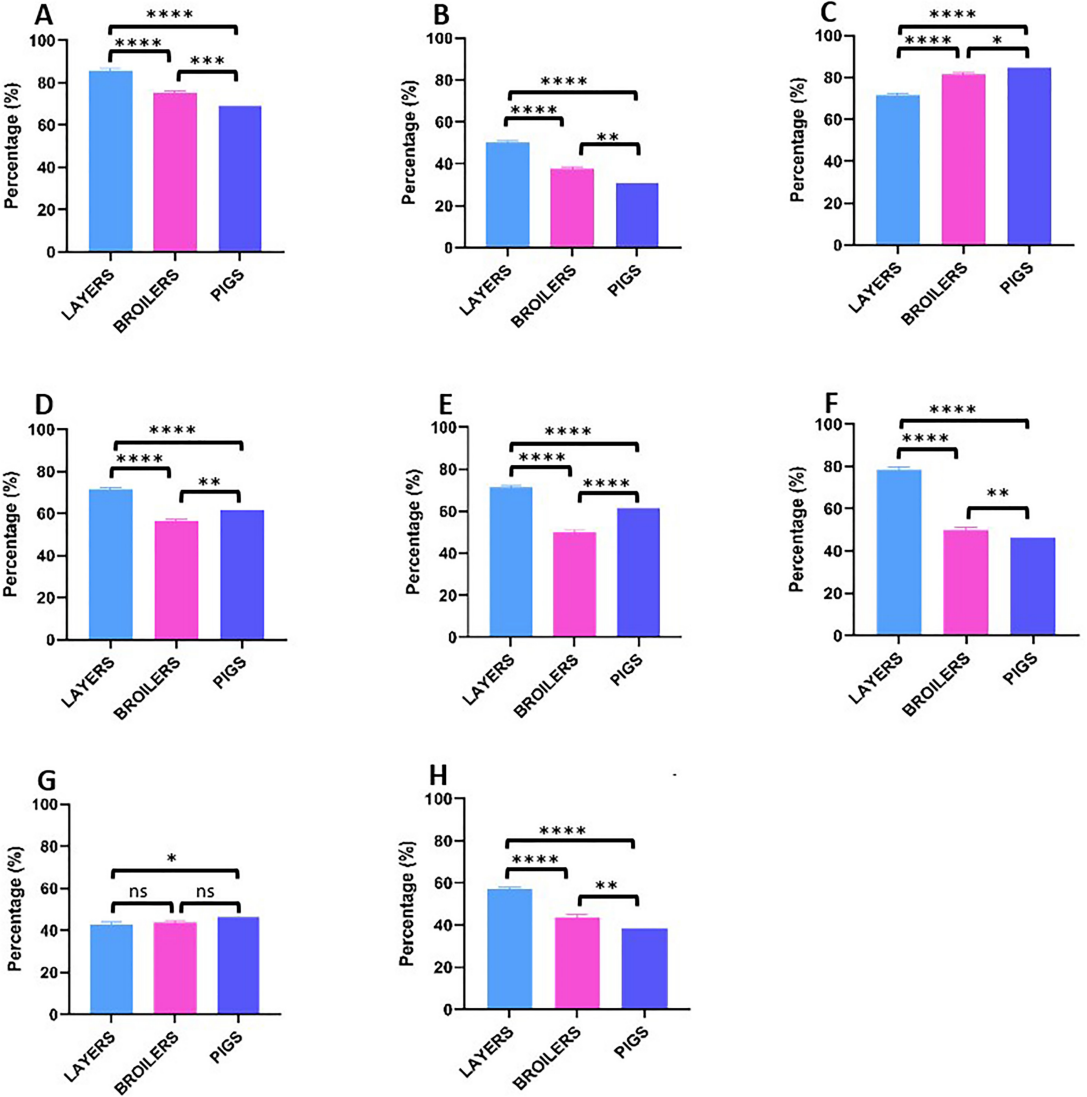
Farm Biosecurity practices by farm type. **(A)** Disinfection and cleaning of Premises. **(B)** Don’t share equipment from other farms. **(C)** Workers using PPE. **(D)** Use of foot dip at farm entrance. **(E)** Quarantine protocol for sick animals. **(F)** Restriction of visitors based on risks. **(G)** Provision of PPEs for visitors. **(H)** Protection of feed from pests and wildlife. promotion. *P*-value significance: ****p < 0.0001; ***p < 0.001; **p < 0.01; *p < 0.05 ns (not significant) *p* > 0.05.

**FIGURE 8:**
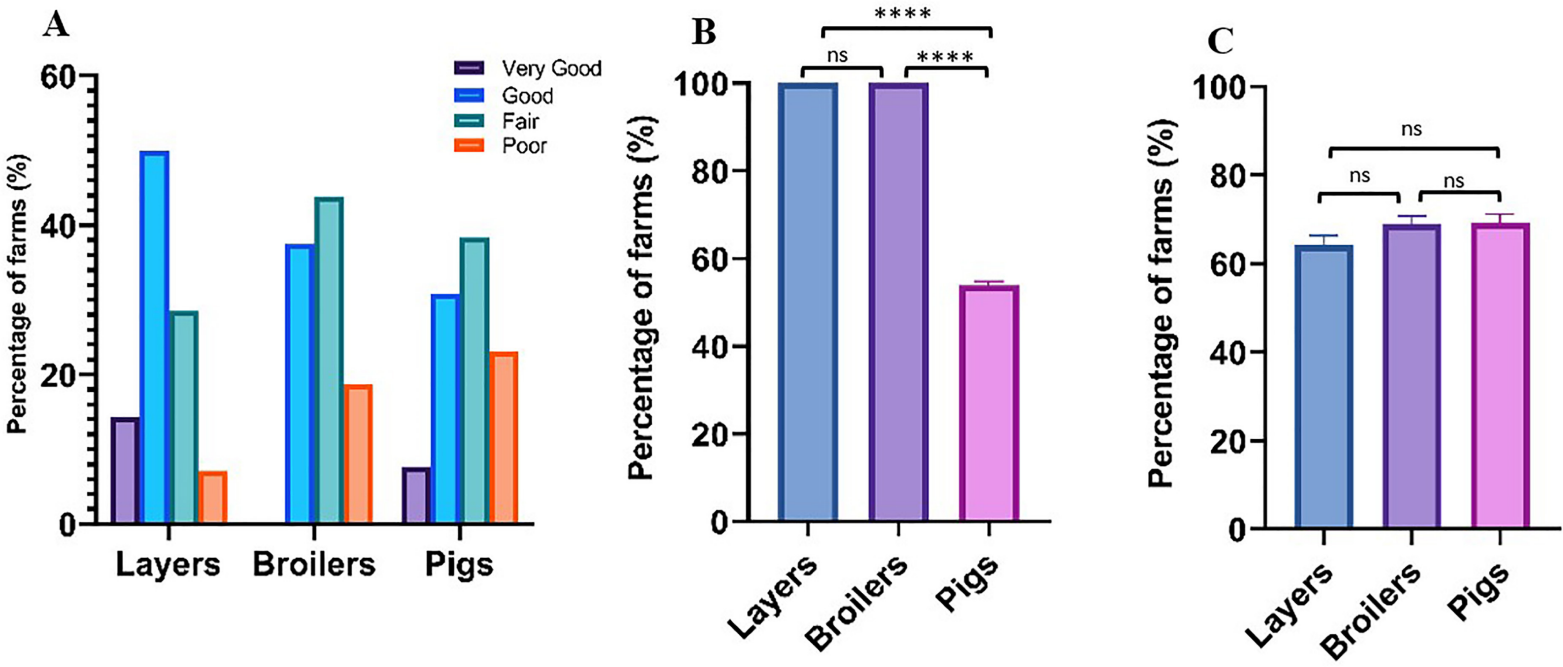
**(A)** Biosecurity Ratings by farm type. **(B)** Vaccination by farm type, **(C)** Proper waste disposal by farm type. *P*-value significance: ****p < 0.0001; ***p < 0.001; **p < 0.01; *p < 0.05 ns (not significant) *p* > 0.05.

Correlation analysis examining the relationship between observed phenotypic resistance in bacterial isolates and documented on-farm antibiotic usage for six overlapping antibiotics revealed moderate positive associations in layer farms (*r* = 0.400, *p* = 0.432) and broiler farms (*r* = 0.487, *p* = 0.327). However, pig farms demonstrated minimal correlation between antibiotic use and resistance patterns (*r* = 0.058, *p* = 0.913), suggesting additional factors beyond direct usage may influence resistance development in swine production systems (Figure 6). Biosecurity assessments revealed substantial variation in standards across operations (Figure 7). Layer farms demonstrated significantly better biosecurity compared to other farm types, with 50% rated as good, 28.6% as fair, and 14.3% as very good. Broiler farms exhibited more variable biosecurity standards: 37.5% good, 43.8% fair, and 18.8% poor. Pig farms showed the most concerning biosecurity profile, with 38.4% rated as fair, 30.8% as good, 23.1% as poor, and only 7.7% as very good (Figure 8). Waste management practices demonstrated moderate consistency across farm types, with proper disposal protocols implemented in 64.3% of layer farms, 62.5% of broiler farms, and 61.5% of pig farms. Vaccination programs were uniformly maintained across layer (100%) and broiler (100%) operations but revealed significant gaps in pig farms (53.8%), potentially contributing to increased prophylactic antimicrobial use and disease susceptibility in swine production systems.

## Discussion

Global surveillance consistently identifies food-producing animals as primary *Salmonella* reservoirs, particularly in sub-Saharan Africa, where antimicrobial resistance (AMR) compounds transmission risks (Aworh et al., 2024). This study reveals a complex relationship between preharvest antibiotic use and AMR in *Salmonella* isolates from commercial poultry and swine farms in Lagos, Southwestern Nigeria. While our study confirms that farms with frequent and unregulated antibiotic use yield resistant *Salmonella* strains, establishing the biological plausibility of this association, the statistical correlations between specific on-farm antibiotic usage and phenotypic resistance patterns proved weaker than anticipated. This apparent inconsistency likely reflects the multifaceted nature of resistance development, where selective pressure from continuous antibiotic exposure intersects with horizontal gene transfer, environmental contamination, and the introduction of resistant strains from external sources (Kumar et al., 2025).

Our study reported a confirmed *Salmonella* prevalence of 1.5% (95% CI: 1.1–1.9%; 54/3,600 samples) across layers, broilers, and pigs in Lagos State, Nigeria, validated through definitive molecular identification protocols that minimized false positives. This population-based rate represents a significant improvement over regional case-series reports documenting 10.8–14.4% prevalence (Waktole et al., 2024; Gobena et al., 2024), likely reflecting their focus on diarrheal episodes and targeted sampling of suspected pathogen sources. However, our findings closely align with systematic surveillance in Ghana (4% poultry) and Tanzania (1% diverse livestock) (Thye et al., 2025), and support Akinyemi et al. (2023), who reported a *Salmonella* prevalence of 2.2% in bovine populations within Lagos Nigeria. Host-specific patterns reveal pigs as an unexpected high-risk reservoir, in contrast to the typical regional dominance of poultry. This divergence likely results from pig productions waste management (liquid manure) systems, which increase environmental contamination compared to poultrys solid waste management. This is further intensified by the direct human-animal contact typical of peri-urban pig farms and by the inadequate waste disposal infrastructure observed in the study. The markedly lower biosecurity standards documented in pig farms relative to poultry operations in this study are of significant concern, not only in the context of *Salmonella* transmission and AMR dissemination, but also in relation to broader biosecurity threats facing the Nigerian swine industry. African Swine Fever (ASF), a highly contagious and economically devastating viral disease with no available vaccine or treatment, poses an existential threat to pig production in West Africa (Ceruti et al., 2025). Suboptimal biosecurity measures including inadequate visitor restrictions, poor waste management, and limited use of personal protective equipment, create conditions highly conducive to the introduction and spread of ASF and other pathogens. Strengthening biosecurity infrastructure in pig farms, including the adoption of standardized protocols for farm entry, disinfection, animal movement control, and surveillance, is therefore a public health and biosecurity imperative that extends well beyond antimicrobial resistance. The European Union’s consistently low prevalence (0–2%) demonstrates that achievable benchmarks can be set through systematic pathogen control programs mandated across livestock systems. Regional rates within SSA, despite gradual diagnostic advances, underscore persistent transmission facilitated by intensive production densities, suboptimal biosecurity, and potentially higher antimicrobial usage patterns versus European standards (Hobbelen et al., 2024). Cross-study variations may also stem from methodological differences, including cohort size and sampling approaches, with our population-based design offering greater generalizability than case-based surveillance.

The antimicrobial susceptibility profiles, as detailed in Table 2, reveal alarming levels of resistance, particularly to tetracycline (75–92.3%), erythromycin (80–100%), and nalidixic acid (46.2–80%) across animal species studied. These findings are corroborated by investigations conducted by Irrazábal et al. (2025) in Paraguay, which documented resistance rates exceeding 70% among *Salmonella* isolates from poultry and swine populations to both tetracycline and nalidixic acid. A complementary study by Aworh et al. (2024) in Nigeria reported similar resistance patterns among *Salmonella* isolates from bovine sources, showing increased phenotypic resistance to tetracycline (77.8%), macrolides (59.3%), and penicillin (40.7%). Furthermore, a recent study characterizing multidrug-resistant *Salmonella* strains reported 95, 75, and 75% resistance to tetracycline, penicillin, and sulfonamides, respectively, among *Salmonella* recovered from pig populations (Zheng et al., 2024). These findings mirror global trends, as reported by a meta-analysis by Wang et al. (2025), which identified tetracycline resistance in over 70% of *Salmonella* isolates from poultry in sub-Saharan Africa, driven by its widespread use in veterinary medicine. The substantial resistance to nalidixic acid, coupled with emerging ciprofloxacin resistance, represents a particularly concerning development, as fluoroquinolone resistance compromises the effectiveness of first-line treatments for invasive salmonellosis in humans. This increases the risk of treatment failure, prolonged illness, and severe clinical outcomes (Totaro et al., 2025). Furthermore, the documented preharvest use of quinolones in Nigerian livestock production likely contributes to this trajectory. While we observed a relatively lower instance of quinolone resistance below the extreme levels reported by Zavari et al. (2025) in Iranian ruminants, the upward trajectory warrants immediate attention. The pronounced resistance to erythromycin, despite its comparatively limited use in veterinary practice, suggests the presence of co-selective mechanisms involving resistance gene transfer, as demonstrated in recent investigations by Zhang et al. (2024), who identified plasmid-mediated horizontal transfer of resistance genes between *Salmonella* species and other members of the *Enterobacteriaceae* family.

The Multiple Antibiotic Resistance Index (MARI) values ranged from 0.2 to 0.9 (Table 3), with specific isolates such as L175-S and B654-S exhibiting resistance to eight distinct antimicrobial agents. Values exceeding 0.2 suggest that isolates originate from high-risk environments with significant antimicrobial exposure (Mir et al., 2022), consistent with the documented widespread preharvest antibiotic use in the surveyed farms. Comparably, Dagah et al. (2024) reported high MARI values (0.6–1.0) among *Salmonella* isolates from “suya” a widely consumed, open-air, ready-to-eat roasted meat product sold across Nigeria, highlighting the direct public health risk posed by the transfer of resistant Salmonella strains from food animal production systems to the point of human consumption without adequate treatment or food safety controls. Their findings surpass observations by Piryaei et al. (2025), who documented maximum MARI values reaching 0.5 among *Salmonella* isolates from broiler chicken populations in Iran. This observation is reinforced by surveillance data from South African poultry populations, which identified universal multidrug resistance (100%) among all *Salmonella* isolates tested (Dlamini et al., 2024), suggesting that extensive antimicrobial resistance in food animal populations may be a regional phenomenon requiring coordinated intervention strategies. Their isolates demonstrated resistance to three or more antimicrobial classes, with predominant resistance patterns observed against erythromycin (62%), tetracycline (59%), and trimethoprim (32%), and corresponding MARI values ranging from 0.33 to 0.78 (Dlamini et al., 2024). The MAR indices recorded in our study suggest possible therapeutic failures and limited effective treatment options for severe farm-to-fork zoonotic infections. The near-complete susceptibility to gentamicin observed in broiler populations (95%) and elevated susceptibility rates in swine (84.6%) represent a critical finding for preserving therapeutic efficacy in human clinical applications, as emphasized within the World Health Organization’s One Health framework for antimicrobial resistance mitigation strategies (WHO, 2024). These observations are corroborated by Zheng et al. (2024), who similarly reported the absence of gentamicin resistance in their study population.

The molecular characterization of AMR genes among *Salmonella* isolates (Figure 1) demonstrates the extensive distribution of resistance determinants within food-producing animals in our study population. The *ant(3″)-Ia* gene, which confers resistance to aminoglycoside antibiotics, including streptomycin, exhibited the highest prevalence across all livestock species (100, 95, and 80.8% in *Salmonella* strains from layers, broilers, and swine), followed by *tetA* (100, 70, and 100% in strains from layers, broilers, and swine), and *Sul1* (75, 80, and 65.4% in strains from layers, broilers, and swine). We found a strong correlation between the presence of the *tetA* gene and phenotypic tetracycline resistance (Figure 2), thus validating the molecular surveillance methods included in our study. The universal detection of *ant(3″)-Ia* genes despite variable streptomycin resistance suggests this resistance mechanism is evolutionarily conserved, possibly due to chromosomal integration or fitness advantages. The elevated prevalence of these resistance genes demonstrates concordance with the observed phenotypic resistance profiles (Table 3), establishing a robust association between genetic determinants and expressed antimicrobial resistance phenotypes. These findings are corroborated by Karim et al. (2025), who detected the universal presence (100%) of the *tetA* gene in all *Salmonella* strains from chicken populations in Bangladesh, with corresponding resistant phenotypes supporting the present observations. Zavari et al. (2025) reported a 65.7% prevalence of the *Sul1* gene among *Salmonella* strains, while Dlamini et al. (2024) documented a 56% prevalence of the *ant(3″)-Ia* gene, demonstrating its conservation among *Salmonella* strains from layer populations, consistent with our findings. The absence of *qnrA*, *qnrC*, and *bla*CTX-M genes across all isolates may suggest limited prevalence of resistance mechanisms against fluoroquinolones and extended-spectrum β-lactam antibiotics within the examined *Salmonella* populations. However, the detection of *qnrB* and *qnrS* genes in broiler (40 and 80%, respectively) and swine (61.5 and 50%, respectively) populations suggests the presence of alternative quinolone resistance mechanisms that may compromise therapeutic efficacy for zoonotic infections. These observations align with findings reported by Jemilehin et al. (2024), who documented *qnrB* (11.1%) and *qnrS* (88.9%) genes among *Salmonella* strains from Nigerian chicken populations, with concurrent absence of the *qnrA* gene. The widespread preharvest use of antimicrobial agents for growth promotion and prophylactic applications in broiler and swine production facilities (Figure 5) presumably contributes to the selective pressure favoring these resistance determinants, as subtherapeutic antimicrobial exposure represents a well-established driver of AMR development and dissemination.

The distribution of virulence genes among *Salmonella* strains recovered from layers, broilers, and pigs provides insights into their pathogenic potential. Virulence determinants, including *iroB* (iron acquisition systems), *pipD* (type III secretion system effector protein), *orfL* (putative virulence factor), and *spiC* (intramacrophage survival mechanisms), were detected at variable frequencies (Figure 3). *Salmonella* strains recovered from broilers demonstrated the highest prevalence for *iroB* (75%), while pig-derived strains had the highest prevalence for *pipD* (92.3%), *orfL* (88.5%), and *spiC* (34.6%), respectively. Our observed prevalence of the *iroB* gene is lower than the documented universal prevalence (100%) of the *iroN* gene (Zavari et al., 2025), an alternative siderophore receptor, which suggests potential strain-specific variation in iron acquisition mechanisms. Interestingly, we observed unique distribution patterns of *Salmonella*-associated virulence genes in strains recovered from swine populations in the study area. It is also notable that swine have significantly longer production cycles than broiler chickens, typically 5–7 months for commercial pigs compared to 5–6 weeks for broilers, resulting in a substantially extended period of cumulative antibiotic exposure per animal. This prolonged selection pressure may in part, explain the higher diversity of resistance gene combinations observed in pig-derived isolates and the broader virulence gene profiles documented for this population. Similarly, layer hens, which may remain in production for 12–18 months, are subject to extended antibiotic exposure than broilers. In our study, the swine population comprised both grower and finisher-stage animals; future investigations may consider stratifying the prevalence of *Salmonella* strains by age and production stage to assess the relationship between duration of antibiotic exposure and resistance accumulation. *Salmonella* strains from these swine populations may possess enhanced host colonization and survival capabilities, thereby amplifying their zoonotic transmission potential. The comparatively lower prevalence of *spiC* (12, 10, and 34.6% in strains from layers, broilers, and swine) suggests that intramacrophage survival mechanisms might not constitute a predominant virulence trait among these strains, possibly limiting their capacity to cause systemic infections in host organisms (Yu et al., 2024). These observations align with findings reported by Dlamini et al. (2024) in their investigation of poultry populations in South Africa, where only 26% of *Salmonella* isolates harbored the *spiC* gene. Furthermore, the heterogeneous distribution of virulence determinants across livestock species as recorded in our study may reflect host-specific evolutionary adaptations or differential selective pressures within distinct agricultural production environments. The intensive production systems typical of broiler and swine operations in the study area may facilitate the selection of *Salmonella* strains harboring comprehensive virulence gene repertoires, as these production environments often impose stressors, including overcrowding and intensive antimicrobial exposure regimens (Li et al., 2024).

Responses from the farm management survey revealed systemic drivers of *Salmonella* persistence and AMR. All surveyed farms were conventional, with most employing intensive production systems (13/14 layer farms, 16/16 broiler farms, and 13/13 pig farms), which are mainly associated with higher pathogen loads due to overcrowding and stress (Kovács et al., 2025). We recorded widespread preharvest use of antibiotics, with tetracycline, ampicillin, and sulfamethoxazole-trimethoprim being most commonly used for animal production. This finding is substantiated by Adebowale et al. (2025), who reported the on-farm use of fluoroquinolones, aminoglycosides, tetracyclines, macrolides, penicillins, and other high-priority critically important antimicrobials in Lagos, Nigeria. Notably, 85–100% of farms in our study reported using antibiotics for treatment, while 53.8–81.3% used them for prophylaxis or growth promotion, particularly in broiler and pig farms. Although only six of the antibiotics used on-farm were included in the susceptibility testing panel, weak positive correlations were observed between on-farm antibiotic use patterns and bacterial AMR phenotypic profiles (*r*- and *p*-values less than 0.5 and greater than 0.05, respectively) (Figure 6). These findings indicate that preharvest antibiotic use does not consistently demonstrate strong correlations with resistance patterns in *Salmonella* recovered from food animals. Our farm management questionnaire included a structured biosecurity assessment module, and the findings revealed that poor biosecurity practices, including infrequent disinfection, equipment sharing across farms, inadequate quarantine of sick animals, and limited visitor restriction, were particularly prevalent in broiler and pig operations (Figure 7). These deficiencies may represent important pathways for the introduction of resistant *Salmonella* strains from external sources, including other farms, feed inputs, water sources, wildlife, and human personnel. The persistence of MDR *Salmonella* independent of direct antibiotic use may therefore partly reflect the continual reseeding of resistant populations through these biosecurity gaps. Consequently, AMR *Salmonella* may persist in food animal populations independent of ongoing antibiotic use, suggesting that resistance patterns are influenced by factors beyond immediate antimicrobial selection pressure. This observation is corroborated by Parzygnat et al. (2025), who demonstrated that the AMR profiles of *Salmonella* strains recovered from backyard chickens raised without antibiotic administration were not statistically significantly different from those obtained from commercial chickens subjected to antibiotic use. Beyond direct on-farm antibiotic use, several inter-related factors may sustain AMR in food animal populations, including: horizontal gene transfer between co-resident bacterial species facilitated by mobile genetic elements such as plasmids and integrons; environmental contamination of soil and water through manure disposal; co-selection of resistance through the use of disinfectants and heavy metals; and the introduction of pre-resistant strains through animal trade, shared inputs, or contaminated feed (Zhang et al., 2024; Odey et al., 2023). These pathways collectively illustrate why resistance profiles may persist even in the absence of ongoing antibiotic pressure and underscore the need for holistic, One Health-oriented surveillance and intervention strategies. Self-prescription of antibiotics was prevalent (71.4–87.5%) across the farms studied, indicating a lack of veterinary oversight that facilitates inappropriate dosing and the selection of resistant strains (Sundram et al., 2024). Statistical analysis revealed significant variations in antibiotic usage patterns and biosecurity practices across different farm types (Figure 7). Layer farms demonstrated significantly superior biosecurity measures compared to other production systems, suggesting a measurable relationship between farm type and the implementation of disease prevention and control strategies. Vaccination programs were universally maintained across layer (100%) and broiler (100%) operations; however, it is important to note that these programs targeted common poultry pathogens (Newcastle disease, infectious bursal disease) and did not include *Salmonella*-specific vaccines, which are not yet standard practice in Nigerian commercial poultry production. Pig farms reported significantly lower vaccination coverage (53.8%), with no *Salmonella*-specific vaccines reported across any animal type. The absence of *Salmonella* vaccination, combined with poor biosecurity in pig operations, may therefore have contributed to the higher *Salmonella* prevalence observed in swine populations and warrants consideration in future disease control strategies. Biosecurity measures varied widely, with 30.8–50% of farms rated as having good biosecurity, while proper waste disposal was reported in 64.3–69.2% of farms, consistent with the 64.7% overall biosecurity compliance reported by Adebowale et al. (2025) in Lagos, Nigeria. These suboptimal practices likely facilitate *Salmonella* transmission between animals, farms, and environments (Chea et al., 2025). These findings underscore the urgent need for strengthened antimicrobial stewardship in Nigerian livestock production, achievable through improved veterinary oversight, enforceable restrictions on non-therapeutic antibiotic use, and targeted farmer education on responsible drug administration. Complementary investments in enhanced surveillance infrastructure are equally essential for monitoring evolving resistance trends, enabling early detection of emerging threats, and informing evidence-based policy decisions at both the national and regional levels.

This study is not without constraints. Reliance on farm-level self-reporting for antibiotic use data introduces the possibility of recall bias and underreporting, while the cross-sectional design precludes causal inference, even where observed associations are mechanistically plausible and globally corroborated. Additionally, *Salmonella* isolates were confirmed to the genus level; serotyping and subspecies-level characterization were not performed, which limits the epidemiological specificity and clinical applicability of the findings. Future work should therefore incorporate expanded molecular analyses—including serotyping and, ideally, whole-genome sequencing—to provide deeper insight into resistance mechanisms, horizontal gene transfer dynamics, pathogenic lineage tracking, and the attribution of resistance determinants to specific circulating serovars. Notwithstanding these constraints, this study offers substantive epidemiological evidence from a setting where integrated data on preharvest antibiotic use and *Salmonella* AMR remain comparatively scarce, thereby addressing a meaningful gap in the regional literature.

## Conclusion

This study reveals a worrisome pattern of antimicrobial resistance in *Salmonella* strains recovered from commercial livestock in Lagos, southwestern Nigeria. Although AMR does not strongly correlate with on-farm antibiotic use, the combination of intensive production systems, poor biosecurity, widespread inappropriate antimicrobial use, and minimal regulatory oversight has created conditions that are ideal for the emergence and spread of resistance. Immediate policy interventions are necessary to mandate veterinary oversight, discourage antibiotic growth promotion practices, improve biosecurity standards, and implement comprehensive monitoring. Without such action, the therapeutic arsenal for treating both animal and human *Salmonella* infections will continue to erode—with potentially severe consequences for food safety and public health in Nigeria and beyond.

## Data Availability

The original contributions presented in the study are included in the article/Supplementary material, further inquiries can be directed to the corresponding author.

## Footnotes

1
https://www.graphpad.com

2
https://jupyter.org
