# Supplementary material for: Preharvest antibiotic use influences antibiotic resistance in Salmonella species from commercial poultry and swine farms in Lagos, Southwestern Nigeria
Source: Front Microbiol. 2026 Apr 28;17:1825884. doi: 10.3389/fmicb.2026.1825884 (PMC13160882; doi:10.3389/fmicb.2026.1825884)
Supplement: Supplementary file 1 [file Table_1.docx]

**Supplementary Table 1:** Targeted primer sequences and PCR conditions for selected virulence, genus-specific, and antimicrobial resistance genes.

| **Target** | **Sequence (5' - 3')** | **Product**  **(bp)** | **PCR Conditions** | | | | **Reference** |
| --- | --- | --- | --- | --- | --- | --- | --- |
|  |  |  | **Denaturation** | **Annealing** | **Extension** | **Cycles** |  |
|  |  |  | $\mathbf{℃}$ **s** | $\mathbf{℃}$ **s** | $\mathbf{℃}$ **s** |  |  |
| invA | invA F: TCATCGCACCGTCAAAGGAACC  invA R: GTGAAATTATCGCCACGTTCGGGCAA | 284 | 95 30 | 58 30 | 72 60 | 34 | (Li et al., 2012; Mthembu et al., 2019) |
| iroB | iroB F: TGC GTA TTC TGT TTG TCG GTCC  iroB R: TAC GTT CCC ACC ATT CTT CCC | 606 | 95 30 | 55 30 | 72 60 | 34 | (Bäumler et al., 1997; Mthembu et al., 2019) |
| spiC | spiC F: CCTGGATAATGACTATTGAT  spiC R: AGTTTATGGTGATTGCGTAT | 309 | 94 60 | 54 30 | 72 60 | 34 | (Hughes et al., 2008; Zishiri et al., 2016) |
| orfL | orfL F: GGAGTATCGATAAAGATGTT  orfL R: GCGCGTAACGTCAGAATCAA | 350 | 94 60 | 58 60 | 72 60 | 35 | (Hughes et al., 2008; Zishiri et al., 2016) |
| pipD | pipD F: CGGCGATTCATGACTTTGAT  pipD R: CGTTATCATTCGGATCGTAA | 350 | 94 25 | 56 30 | 72 50 | 34 | (Hughes et al., 2008; Zishiri et al., 2016) |
| qnrA | qnrA F: CCGCTTTTATCAGTGTGACT  qnrA R: ACTCTATGCCAAAGCAGTTG | 188 | 95 30 | 55 30 | 72 30 | 35 | (Herrera-Sánchez et al., 2021) |
| qnrB | qnrB F: GATCGTGAAAGCCAGAAAGG  qnrB R: ACGATGCCTGGTAGTTGTCC | 469 | 95 30 | 54 30 | 72 30 | 35 | (Herrera-Sánchez et al., 2021) |
| qnrC | qnrC F: GGGTTGTACATTTATTGAATCG  qnrC R: CACCTACCCATTTATTTTCA | 308 | 95 30 | 54 30 | 72 30 | 35 | (Herrera-Sánchez et al., 2021) |
| qnrS | qnrS F: ACGACATTCGTCAACTGCAA  qnrS R: TAAATTGGCACCCTGTAGGC | 417 | 95 30 | 55 30 | 72 30 | 35 | (Herrera-Sánchez et al., 2021) |
| *Sul*1 | *Sul*1 F: GCG CGG CGT GGG CTA CCT  *Sul*1 R: GATTTCCGCGACACCGAGACAA | 350 | 94 25 | 65 30 | 72 50 | 34 | (Zishiri et al., 2016) |
| *bla*_CTX-M | *bla*_CTX-M F: ATGTGCAGYACCAGTAARGT  *bla*_CTX-M R: TGGGTRAARTARGTSACCAGA | 593 | 96 60 | 60 60 | 72 60 | 30 | (Farzi et al., 2021) |
| ant (3”)-la | ant (3”)-la F: GTGGATGGCGGCCTGAAGCC  ant (3”)-la R: ATTGCCCAGTCGGCAGCG | 526 | 94 60 | 58 60 | 72 60 | 35 | (Zishiri et al., 2016) |
| tetA | tetA -F: GCTACATCCTGCTTGCCTTC  tetA -R: CATAGATCGCCGTGAAGAGG | 210 | 94 25 | 55 30 | 72 50 | 34 | (Zishiri et al., 2016) |
