# Supplementary material for: Preharvest antibiotic use influences antibiotic resistance in Salmonella species from commercial poultry and swine farms in Lagos, Southwestern Nigeria
Source: Front Microbiol. 2026 Apr 28;17:1825884. doi: 10.3389/fmicb.2026.1825884 (PMC13160882; doi:10.3389/fmicb.2026.1825884)
Supplement: Supplementary file 2 [file Table_2.docx]

**A**


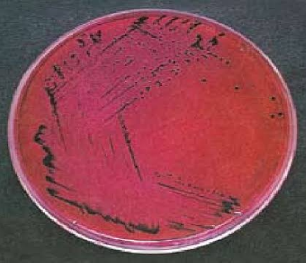

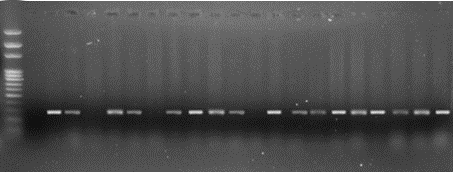


**L NC S1 S2 S3 S4 S5 S6 S7 S8 S9 S10 S11 S12 S13 S14 S15 S16 S17 S18 S19 S20**

Lane 1: 100bp Molecular ruler

Lane 2: Negative control

Lane 3-12: amplicons from PCR.

**284bp**

**B**

**Supplementary Figure 1: A:** Representative image from the culture of *Salmonella* on XLD. **B:** Representative Gel image for Genus-specific (*invA)* gene PCR for *Salmonella* isolates

**C**


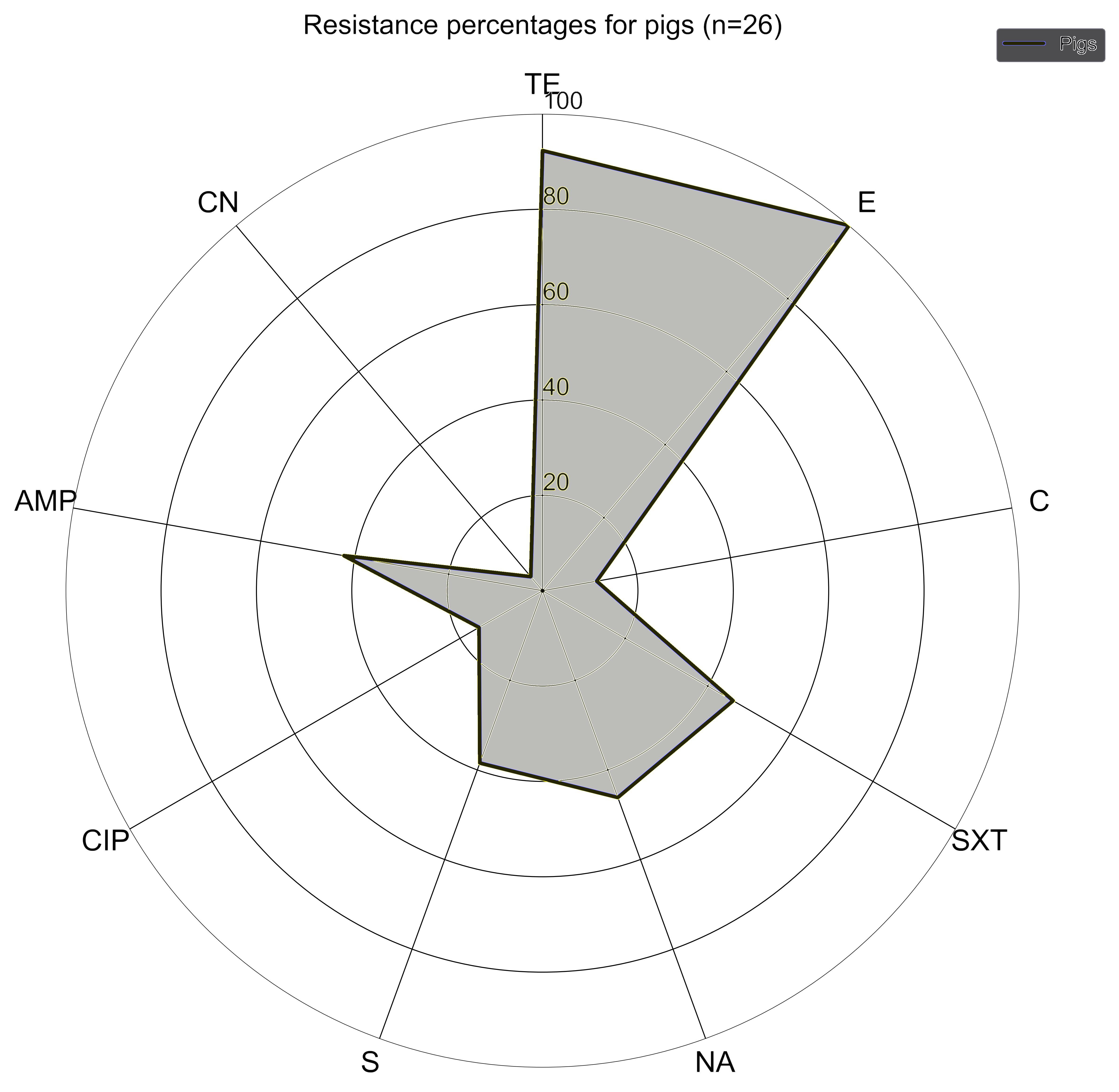


**B**


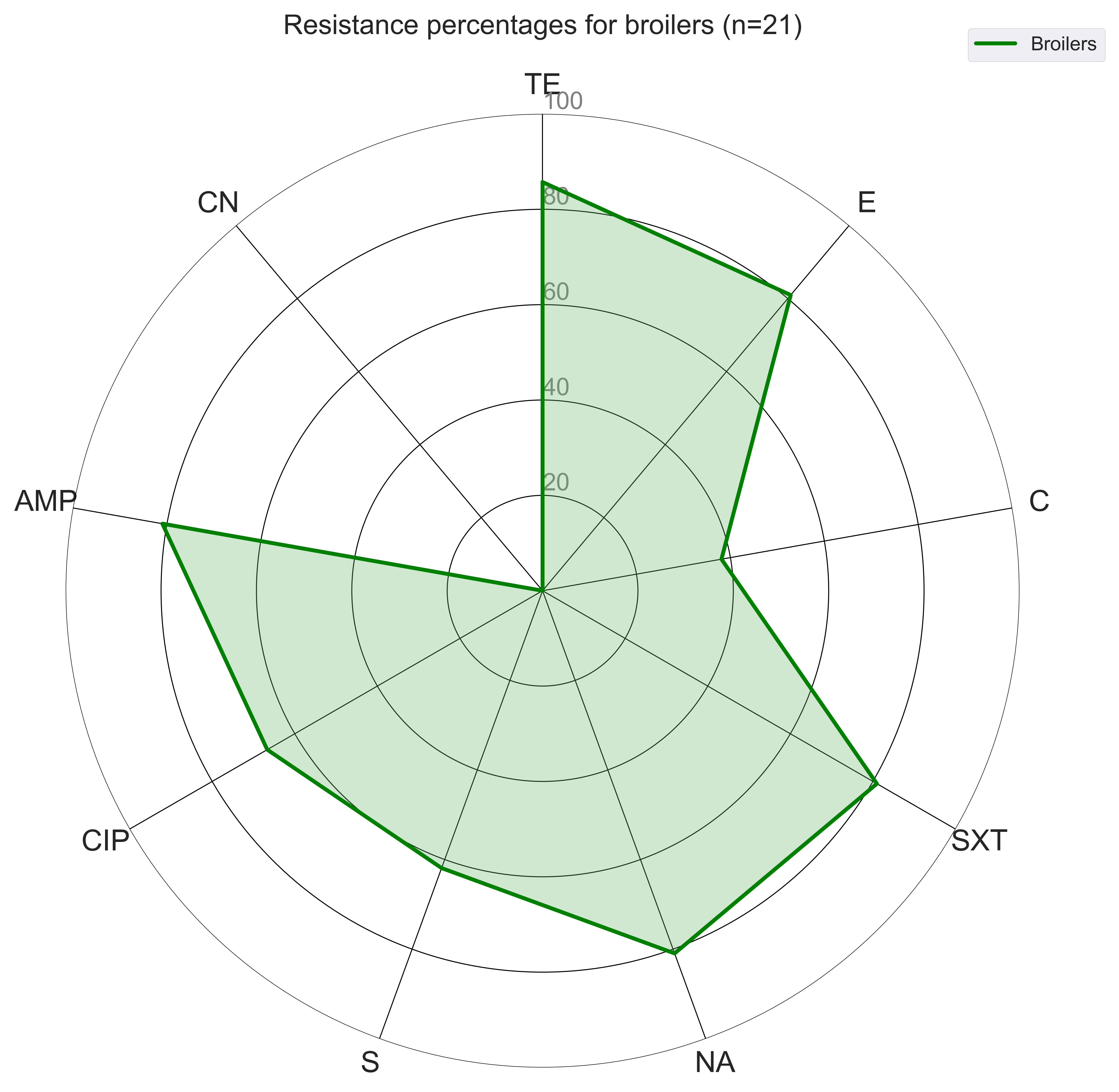


**A**


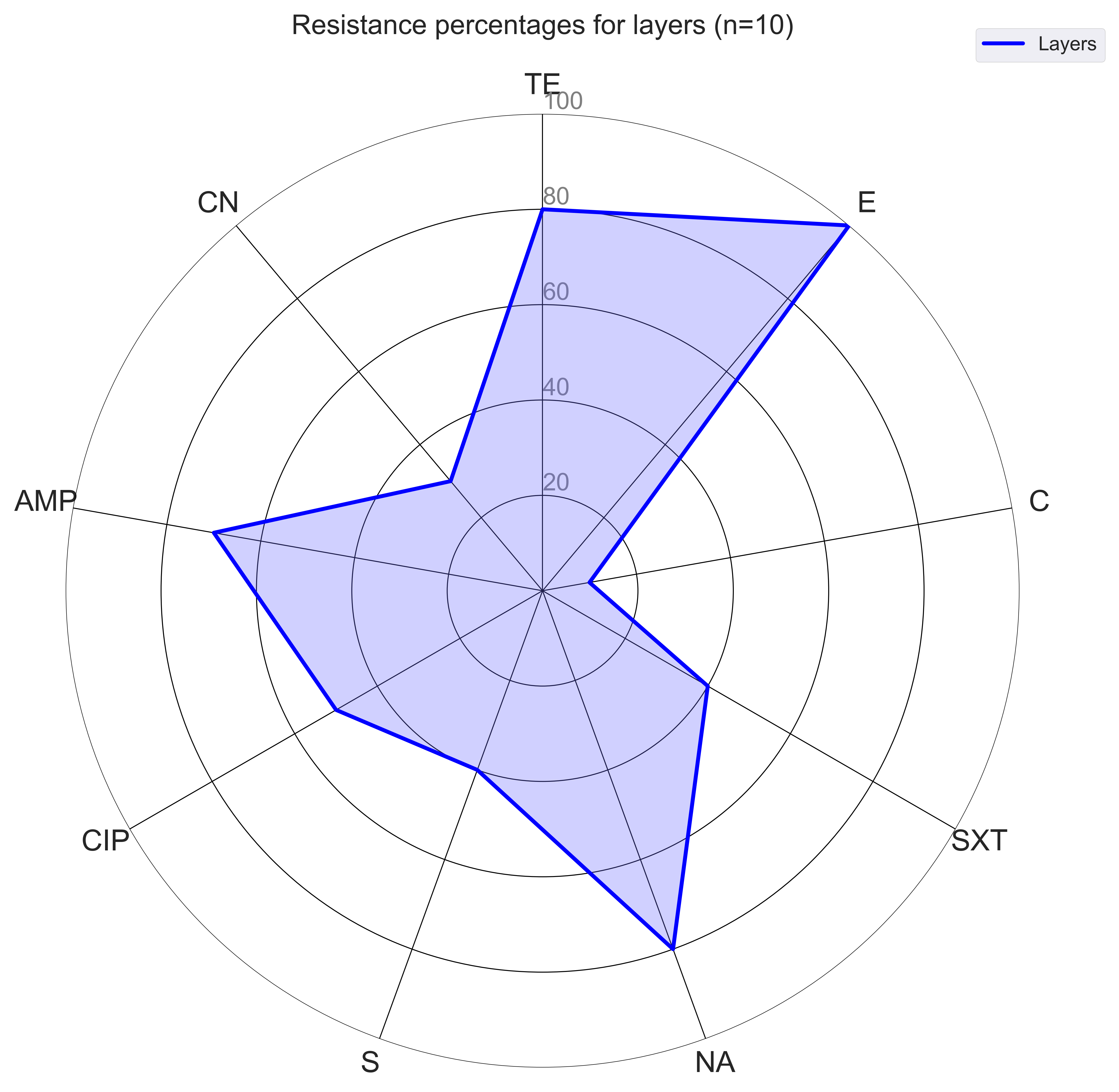


**Supplementary Figure 2:** Resistance pattern of Salmonella from A: layers, B: broilers, and C: pigs

**TE**: tetracycline, **E**: erythromycin, **C**: chloramphenicol, **SXT**: sulfamethoxazole-trimethoprim, **NA**: nalidixic acid, **S**: streptomycin, **AMP**: ampicillin, **CN**: gentamicin, **CIP:** ciprofloxacin.


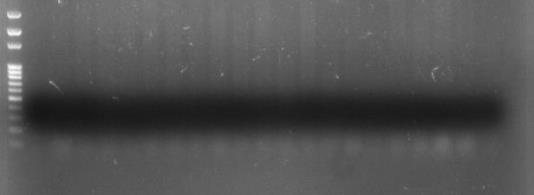


**L NC S1 S2 S3 S4 S5 S6 S7 S8 S9 S10 S11 S12 S13 S14 S15 S16 S17 S18 S19 S20**

Lane 1: 100bp Molecular ruler

Lane 2: Negative control

Lane 3-12: amplicons from PCR.

**593bp**

**D**

**B**


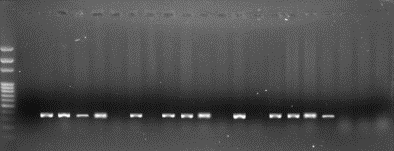


**L NC S1 S2 S3 S4 S5 S6 S7 S8 S9 S10 S11 S12 S13 S14 S15 S16 S17 S18 S19 S20**

Lane 1: 100bp Molecular ruler

Lane 2: Negative control

Lane 3-12: amplicons from PCR.

**350bp**

**A**


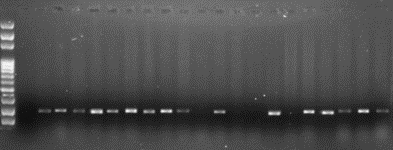


**L NC S1 S2 S3 S4 S5 S6 S7 S8 S9 S10 S11 S12 S13 S14 S15 S16 S17 S18 S19 S20**

Lane 1: 100bp Molecular ruler

Lane 2: Negative control

Lane 3-12: amplicons from PCR.

**210bp**


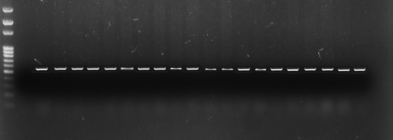


**L NC S1 S2 S3 S4 S5 S6 S7 S8 S9 S10 S11 S12 S13 S14 S15 S16 S17 S18 S19 S20**

Lane 1: 100bp Molecular ruler

Lane 2: Negative control

Lane 3-12: amplicons from PCR.

**526bp**

**C**

**E**


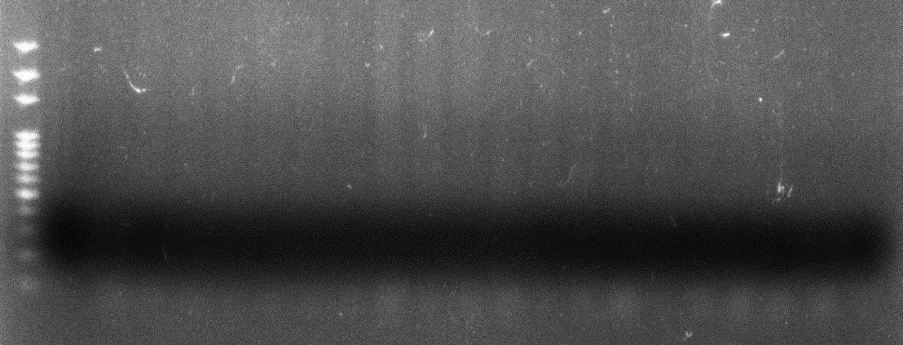


**L NC S1 S2 S3 S4 S5 S6 S7 S8 S9 S10 S11 S12 S13 S14 S15 S16 S17 S18 S19 S20**

Lane 1: 100bp Molecular ruler

Lane 2: Negative control

Lane 3-12: amplicons from PCR.

**188bp**

**F**


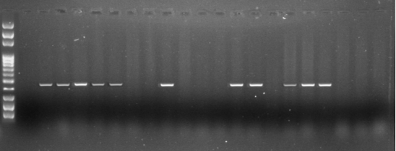


**L NC S1 S2 S3 S4 S5 S6 S7 S8 S9 S10 S11 S12 S13 S14 S15 S16 S17 S18 S19 S20**

Lane 1: 100bp Molecular ruler

Lane 2: Negative control

Lane 3-12: amplicons from PCR.

**469bp**


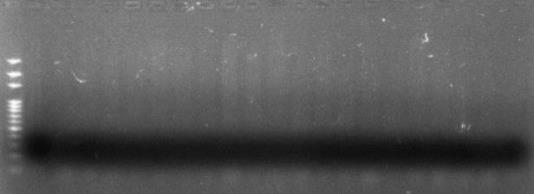


**L NC S1 S2 S3 S4 S5 S6 S7 S8 S9 S10 S11 S12 S13 S14 S15 S16 S17 S18 S19 S20**

Lane 1: 100bp Molecular ruler

Lane 2: Negative control

Lane 3-12: amplicons from PCR.

**308bp**

**G**


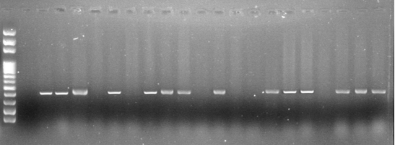


**L NC S1 S2 S3 S4 S5 S6 S7 S8 S9 S10 S11 S12 S13 S14 S15 S16 S17 S18 S19 S20**

Lane 1: 100bp Molecular ruler

Lane 2: Negative control

Lane 3-12: amplicons from PCR.

**417bp**

**H**

**Supplementary Figure 3:** Representative Gel images for *tetA* **(A)***, sul1* **(B)**, *ant-3(‘’)-la* **(C)**, *bla_CTX-M* **(D)**, *qnrA* **(E)**, *qnrB* **(F)*,*** *qnrC* **(G)**, and *qnrS* **(H)** genes PCR for *Salmonella* isolates


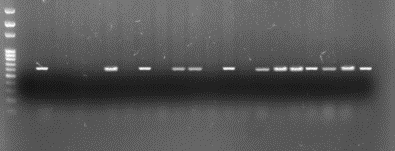


**L NC S1 S2 S3 S4 S5 S6 S7 S8 S9 S10 S11 S12 S13 S14 S15 S16 S17 S18 S19 S20**

Lane 1: 100bp Molecular ruler

Lane 2: Negative control

Lane 3-12: amplicons from PCR.

**606bp**

**A**

**B**


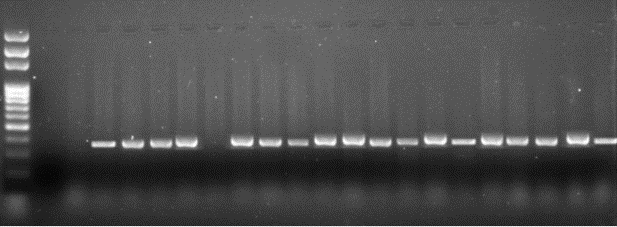


**L NC S1 S2 S3 S4 S5 S6 S7 S8 S9 S10 S11 S12 S13 S14 S15 S16 S17 S18 S19 S20**

Lane 1: 100bp Molecular ruler

Lane 2: Negative control

Lane 3-12: amplicons from PCR.

**350bp**


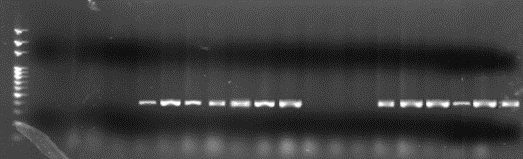


**L NC S1 S2 S3 S4 S5 S6 S7 S8 S9 S10 S11 S12 S13 S14 S15 S16 S17 S18 S19**

Lane 1: 100bp Molecular ruler

Lane 2: Negative control

Lane 3-12: amplicons from PCR.

**350bp**

**C**


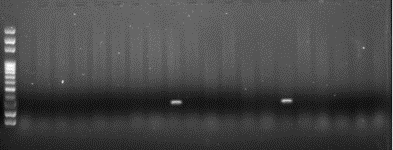


**L NC S1 S2 S3 S4 S5 S6 S7 S8 S9 S10 S11 S12 S13 S14 S15 S16 S17 S18 S19**

Lane 1: 100bp Molecular ruler

Lane 2: Negative control

Lane 3-12: amplicons from PCR.

**309bp**

**D**

**Supplementary Figure 4:** Representative Gel images for *iroB* **(A),** *pipD* **(B),** *orfL* **(C),** and *spiC* **(D)** genes PCR for *Salmonella* isolates


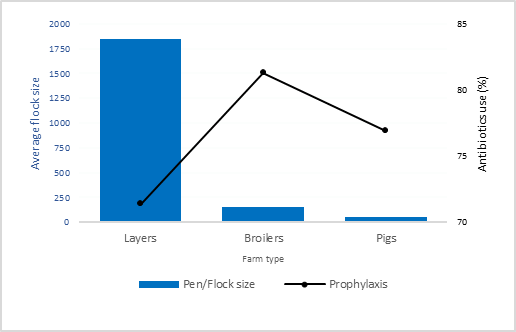


**B**


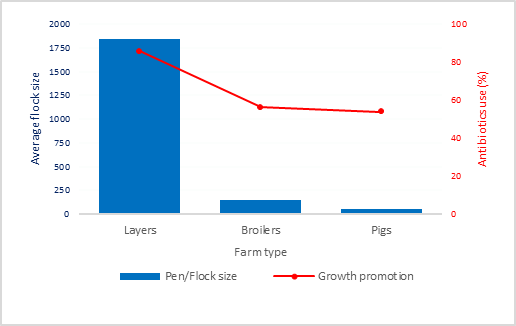


**A**


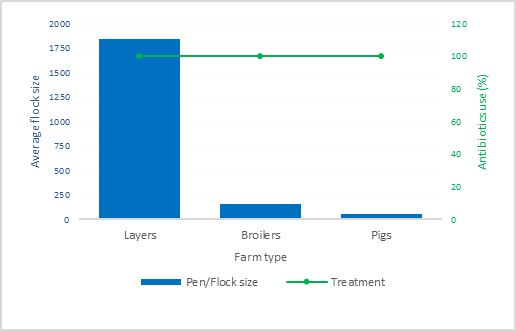


**C**

**Supplementary Figure 5:** Comparison of flock size to antibiotic usage: **A.** Growth promotion, **B.** Prophylaxis, **C.** Treatment
