# Supplementary material for: Preharvest antibiotic use influences antibiotic resistance in Salmonella species from commercial poultry and swine farms in Lagos, Southwestern Nigeria
Source: Front Microbiol. 2026 Apr 28;17:1825884. doi: 10.3389/fmicb.2026.1825884 (PMC13160882; doi:10.3389/fmicb.2026.1825884)
Supplement: Supplementary file 3 [file Table_3.docx]

**Farm Manager Questionnaire**

Characterization of major zoonotic bacteria from food animals in Lagos state, Nigeria.

Farm ID:

Farm Location:

Dear Farmer,

**What is the project about?**

The project is centered on determining and characterizing the major zoonotic pathogens in Lagos State, Nigeria. This study would provide information and an in-depth understanding of the animal farm characteristics and biosecurity practices, as well as the level of on-farm antibiotic use in Lagos State, Nigeria. Furthermore, we hope to estimate the baseline flock-level prevalence of these zoonotic pathogens in Lagos, Nigeria, through the collection of fecal samples from your farm.

**Why are we carrying out the project?**

This study proffers a novel approach to the issue of global drug resistance in-country by determining the prevalence of major zoonotic pathogens and the level of antimicrobial resistance while offering safer alternatives for farmers in Nigeria. The characterization of these zoonotic pathogens will inform the right scientific decision necessary for abating the hazards of diseases and drug resistance.

**Benefits of the project to farmers?**

This project offers our farmers healthier and affordable alternatives to conventional antibiotics for treatment, prophylaxis, and growth promotion, which consequently cuts down the risk of antimicrobial resistance in their farms.

**What happens to my data?**

All information gathered will be treated as highly confidential and will only be used for academic and research purposes. Please note, the questionnaire should take only a few minutes of your time and is voluntary. Please ensure you tick all relevant boxes and answer the questions appropriately.

Thank you for your time.

**Signed Informed Consent:**

Signature……………………………………..

**SECTION A: Farm Information**

1. Farm ID: ________________
2. Senatorial District:
   - Lagos West [ ]
   - Lagos East [ ]
   - Lagos Central [ ]
3. Farm Type:
   - Conventional farm [ ]
   - Organic farm [ ]
4. Type of Operation:
   - Poultry (Layers) [ ]
   - Poultry (Broilers) [ ]
   - Pig farming [ ]
5. Pen/Flock Size: ________________ (number of animals)

**SECTION B: Antimicrobial Use**

1. Do you use antibiotics for the treatment of diseases?
   - Yes [ ]
   - No [ ]
2. Do you use antibiotics for prophylaxis (disease prevention)?
   - Yes [ ]
   - No [ ]
3. Do you use antibiotics for growth promotion on your farm?
   - Yes [ ]
   - No [ ]
4. Which antimicrobials do you use on your farm? **(Check all that apply)**
   - [ ] Tetracycline (TE)
   - [ ] Erythromycin (E)
   - [ ] Chloramphenicol (C)
   - [ ] Sulfamethoxazole-trimethoprim (SXT)
   - [ ] Nalidixic acid (NA)
   - [ ] Streptomycin (S)
   - [ ] Ampicillin (AMP)
   - [ ] Gentamicin (CN)
   - [ ] Ciprofloxacin (CIP)
   - [ ] Colistin (CO)
   - [ ] Enrofloxacin (EN)
   - [ ] Amoxicillin (AMX)
   - [ ] Tylosin (TY)
5. Are there other classes of antibiotics used on your farm not listed above? If yes, kindly state them. ________________
6. Who prescribes drugs on your farm?
   - Self-prescription [ ]
   - Veterinary expert [ ]
   - Both self and expert [ ]

**SECTION C: Farm Biosecurity Practices**

1. Do you have proper waste disposal practices in place (e.g., for manure, carcasses, or other waste)?
   - Yes [ ]
   - No [ ]
2. Do you follow a vaccination program for your animals?
   - Yes [ ]
   - No [ ]
3. Do you have foot dip disinfectant at entrances to the farm?

- Yes [ ]
- No [ ]

1. Do you get farm equipment from other farms?

- Yes [ ]
- No [ ]

1. Does the farm have a quarantine protocol for newly introduced animals?

- Yes [ ]
- No [ ]

1. Do you often clean and disinfect farm premises?

- Yes [ ]
- No [ ]

1. Do you restrict visitor access based on the risk they present?

- Yes [ ]
- No [ ]

1. Are visitors provided boots and overalls by you?

- Yes [ ]
- No [ ]

1. Are farmers and workers provided with personal protective equipment (boots, gloves, overalls, etc.)?

- Yes [ ]
- No [ ]

1. Are feeds protected from pests and wildlife?

- Yes [ ]
- No [ ]

1. How would you rate the farm's biosecurity measures following responses from Section C 14-21?
   - Very Good (7-8/8) [ ]
   - Good (5-6/8) [ ]
   - Fair (3-4/8) [ ]
   - Poor (0-2/8) [ ]

**Additional Information:** any additional comments about your farm management practices:
